# *FMR1* gene therapy restores translationally relevant phenotypes in a mouse model for fragile X syndrome

**DOI:** 10.1038/s41434-026-00630-4

**Published:** 2026-07-10

**Authors:** Richard K. Lacher, Kari Henson, Lindsay N. Wathen, Caitlin Jones, Tiffany Arnold, McKenzie R. Rice, Heather M. Carles, Angela R. White, Elizabeth Ramsuchit, Darren Murrey, Rebecca Raig, Austen Fisher, Kaitlin Bucher, Grace C. Westerkamp, Adam L. Fritz, Brooke M. Gollaway, Sebastian Piloto, David Dismuke, J Elliott Robinson, Michael T. Williams, Charles V. Vorhees, Erandi K. De Silva, Durgesh Tiwari, Craig A. Erickson, Ernest V. Pedapati, Christina Gross

**Affiliations:** 1 Division of Child and Adolescent Psychiatry, Cincinnati Children’s Hospital Medical Center, Cincinnati, OH USA; 2 Forge Biologics, Grove City, OH USA; 3 Division of Neurology, Cincinnati Children’s Hospital Medical Center, Cincinnati, OH USA; 4 Division of Experimental Hematology and Cancer Biology, Cincinnati Children’s Hospital Medical Center, Cincinnati, OH USA; 5 Department of Pediatrics, University of Cincinnati College of Medicine, Cincinnati, OH USA; 6 Department of Psychiatry, University of Cincinnati College of Medicine, Cincinnati, OH USA

**Keywords:** Neuroscience, Molecular biology

## Abstract

Fragile X Syndrome (FXS) is the most common inherited form of intellectual disability. It is caused by a trinucleotide expansion in the 5’ UTR of the *Fragile X messenger ribonucleoprotein 1* (*FMR1*) gene leading to loss of expression of Fragile X messenger ribonucleoprotein (FMRP). There is currently no cure for FXS. We developed an *FMR1* gene therapy based on an adeno-associated viral vector designed with strong translational potential for future clinical testing. The viral vector was tested in *Fmr1* knockout mice using two translationally relevant delivery routes and ages corresponding to *in utero*, toddler, and adolescent ages in humans. Functional studies showed that the *FMR1* gene therapy improved select translational FXS phenotypes spanning three critical domains: sensory hyperexcitability, adaptation to change, and altered brain activity. Expression after intracerebroventricular injection was most prominent in the forebrain, whereas intravenous delivery predominantly led to expression across midbrain and brainstem, suggesting that a dual route may be needed to achieve full brain coverage. Biodistribution analyses further suggested that FMRP expression must be titrated carefully for optimal rescue. In summary, we show that *FMR1* gene therapy using delivery routes and vehicles approved for clinical use improves core phenotypes in a mouse model for FXS.

**Figure:**
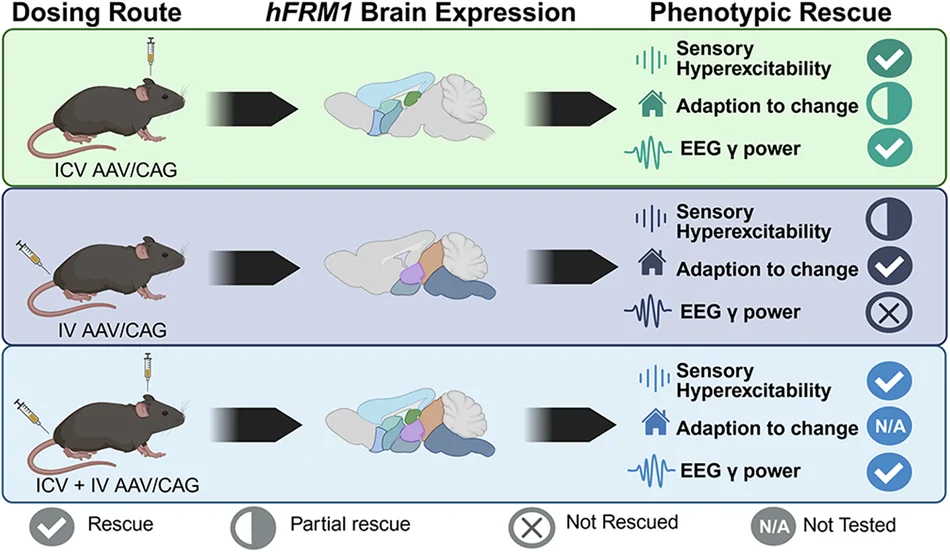

## Introduction

Fragile X Syndrome (FXS) is the most common genetic form of intellectual disability and one of the major monogenic disorders associated with autism. Current clinical management is mainly symptomatic, and no cure or efficient disease-targeted treatment is available. FXS is caused by insufficient levels of fragile X messenger ribonucleoprotein (FMRP). FMRP acts in the soma, nucleus, and synapses, and regulates a plethora of cellular processes, including mRNA editing, stability and translation, ion channel activity, and DNA damage repair [1–3]. About twenty different single-drug rescue strategies have been tested in FXS mice in hundreds of studies so far [4]. Positive results in preclinical models have not translated to successful drug approvals in human clinical trials, despite strong theoretical underpinnings [5]. A small-molecule strategy may be inherently challenged as it may not comprehensively address the many defects caused by the absence of FMRP. The most promising approach for the rescue of FXS phenotypes is to reintroduce FMRP. The *FMR1* gene is hypermethylated and transcriptionally silenced due to a trinucleotide repeat expansion in the 5’UTR in FXS. Notably, expression of low levels of FMRP in patients with mosaicism in the *FMR1* repeat length leads to improved cognition [6] and peripheral FMRP levels are positively correlated with IQ [7, 8], justifying therapeutic approaches to re-express FMRP.

One strategy is to reactivate the *FMR1* gene through reversing hypermethylation [9, 10]; however, this approach will lead to the expression of mRNA containing the 5’ trinucleotide expansion, which could be toxic by itself and induce health issues as seen in premutation carriers [11–13]. A few studies have also attempted to correct the expansion using CRISPR/Cas9 gene editing but this has only been done in vitro in iPSCs and embryonic stem cells so far [14] and without a clear strategy to operationalize gene editing in the human brain.

Another approach is to replace FMRP using adeno-associated viral (AAV) strategies. Of note, AAV gene delivery strategies are increasingly evaluated for the treatment of CNS disorders [15]. Indeed, several preclinical studies in mice suggest that an adeno-associated viral (AAV) gene therapy to re-express FMRP has therapeutic potential [16–19]. However, most of these studies used viral vectors or dosing strategies not readily translatable into human studies: e.g., they re-expressed the mouse *Fmr1* gene [17], used promoters not yet approved for humans [19], and employed phenotypical readouts with low translational potential.

The goal of this study was to preclinically test the efficacy of an *FMR1* gene therapy with strong translational potential. To this end, we chose AAV serotypes and promoter combinations that have already been approved for clinical trials in humans in other indications and included efficacy tests with strong face validity between the mouse model and patients with FXS. Our results show that a human *FMR1* gene therapy improved specific phenotypes with high translational value in *Fmr1* KO mice, namely sensory hyperactivity, stereotypic behavior, and increased EEG gamma power.

## Materials and methods

### Mice

*Fmr1* knockout (KO) mice maintained on a C57BL/6J background (RRID:IMSR_JAX:003025) and C57BL/6J wild-type (WT, RRID:IMSR_JAX:000664) mice were obtained from The Jackson Laboratory (Bar Harbor, ME, USA). Audiogenic seizures in mice injected with virus at 5 weeks of age were done using *Fmr1* KO mice on FVB/NJ background directly purchased from *The Jackson Laboratory* (RRID:IMSR JAX:004624). The *Fmr1* gene is on the X chromosome, male mice are hemizygous but will be named *Fmr1* KO throughout the manuscript. Depending on the study, male and female *Fmr1* KO mice or WT mice were purchased directly from *The Jackson Laboratory* (biodistribution and multi-electrode array (MEA) analyses), generated using *Fmr1* KO x *Fmr1* KO and WT x WT breeding pairs at CCHMC (audiogenic seizures in mice injected with virus at postnatal days 1–3) or generated from *Fmr1* heterozygous/WT breeding pairs to obtain male *Fmr1* KO and WT littermates (mice for behavioral analyses). Mice were used from postnatal day 1 to 120 depending on the assay as indicated in the respective manuscript sections and/or figures. Mice were housed in groups of up to 4 on a 14:10 light-dark cycle with food and water ad lib. All mouse work was approved by the Institutional Animal Care and Use Committees of CCHMC and *Melior Discovery, Inc*. (Exton, PA, USA).

### FMR1 viral vectors and drugs

Three different viral vectors expressing the protein-coding region of human *FMR1* isoform 1 were manufactured at *Forge Biologics* (Grove City, OH, USA) with an AAV9 capsid serotype. Vectors were produced using triple plasmid transfection in a suspension mammalian cell culture. Plasmids were produced by *Aldevron* (Fargo, ND, USA) and *Forge Biologics* (Grove City, OH, USA). Viral titer was determined using ddPCR. Viral vector doses were determined based on current AAV clinical trials and scaled to mouse weights. Doses varied and are indicated under each experiment. Vehicle was PBS with 0.001% Pluronic. Anti-CD20 was purchased from *InvivoGen* (cat. # mcd20-mab10-1, San Diego, CA, USA) and rapamycin was purchased from *Thermo Fisher Scientific* (Waltham, MA, USA).

### Primary neuron culture preparation and transduction

Primary cortical neurons were prepared from *Fmr1* KO mice at embryonic day 16.5–18.5 as described [20], transduced with 0.3–1.2 × 10^10^ viral vector genomes (vg/ml) at 7 days in vitro (DIV), and analyzed by western blot 7 days later.

### Intracerebroventricular injections in adolescent mice

Intracerebroventricular (ICV) injections in adolescent mice were done as described [21]. Briefly, 3–8-week-old WT or *Fmr1* KO mice received bilateral intracerebroventricular (ICV) injections under isoflurane anesthesia using an automated stereotaxic device and the following coordinates from bregma, for 3-week-old mice: anteroposterior (AP) = −0.2 mm; mediolateral (ML) = ±1.0 mm and dorsoventral (DV) = −1.7 mm; for 5-week-old mice: AP = −0.4 mm; ML = ±1.0–1.5 mm and DV = −3 mm; for 7-to-8-week-old mice: AP = −0.3 mm; ML = ±1.0 mm and DV = −2 mm. A 5 µl Neuros^TM^ syringe with beveled needle (cat. # 65460-03, *Hamilton Company*, Reno, NV, USA) was used for injections and was left in place for 5–10 min followed by slow retraction. The skin incision was closed with surgical sutures (*Covidien Medtronic*, Minneapolis, MN, USA) and GLUture (*Zoetis Inc*., Kalamazoo, MI, USA), or with VetBond tissue adhesive (3M, Hebron, KY, USA) followed by application of antibiotic ointment (*RARO*, Hawthorne, NY, USA) on top to prevent infections. To facilitate recovery, mice were placed on a heating pad, injected with 1 ml of sterile Ringer’s saline intraperitoneally (i.p.) and monitored until recovery. Carprofen was given before and after surgery to help with pain management. Viral vector doses were 3 × 10^10^ vg/ventricle (for biodistribution and audiogenic seizures in mice injected at 5 weeks of age), 3.6 × 10^9^ vg/ventricle (low dose) and 2.2 × 10^10^ vg/ventricle (high dose) (for behavioral studies) or 4 × 10^9^ vg/ventricle (low dose) and 2 × 10^10^ vg/ventricle (high dose) (for MEA studies). Doses were adjusted based on clinical studies, aiming for ~2 × 10^10^ vg/g brain mass (low dose) and 1 × 10^11^ vg/g brain mass (high dose), assuming an average brain weight of ~0.39 g for 3-week-old, and ~0.4 g for 6-week-old mice [22].

### Intracerebroventricular injections in neonatal mice

Mice on postnatal days 1–3 were anesthetized by hypothermia using wet ice and placed on a pre-cooled stage for injections. Mice were injected bilaterally using a Hamilton syringe, an automated stereotaxis and the following coordinates from lambda: AP = +1.5 mm; ML = ±0.8 mm and DV = −1.5 mm. After injection of 1 µl of viral vector, the needle was left in place for 1 min and then slowly retracted. Following the surgery, the pup was warmed up by holding it in the hand and then placed on a heated Napa Nectar pack (*Thermo Fisher Scientific*) covered with paper towels. As soon as the pup’s skin tone and activity returned to normal it was placed back in the dam’s cage. Viral vector doses were 6 × 10^9^ vg/ventricle.

### Intravenous tail vein injection in adolescent mice

For initial biodistribution studies, intravenous (IV) injections were done via the tail vein. Briefly, mice were placed in a restrainer and the tail vein visualized. The injection site was swabbed with a disinfectant, and a needle was inserted into the tail vein. The viral vector or vehicle was administered slowly, ensuring injection into the tail vein. The needle was removed after the substance was administered and gentle pressure was applied to the injection site to provide hemostasis. The mice were returned to their cage following injection. IV viral vector dose was 3 × 10^13^ vg/kg. The maximum volume did not exceed 5 ml/kg (125 μl/25 g mouse).

### Intravenous retro-orbital injection in neonatal and adolescent mice

For behavioral studies, intravenous delivery occurred via retro-orbital injections. Mice between 3 and 8 weeks of age were placed in an isoflurane chamber to induce anesthesia. A drop of ophthalmic anesthetic was applied to the eye dedicated for the injection. The viral vector, vehicle, or anti-CD20 antibody was administered slowly to one eye, ensuring that it was injected into the retro-orbital sinus. The needle was removed, and gentle pressure was applied to the injection site to provide hemostasis. The mouse was returned to its cage following injection. For combination IV + ICV dosing, IV injections were done the same day as the ICV injections. The maximum volume did not exceed 150 µl per eye per day. IV viral vector doses were 2.5 × 10^13^ vg/kg (low dose for behavioral studies) and 1 × 10^14^ vg/kg (high dose behavioral studies), or 1 × 10^13^ vg/kg (low dose MEA studies) and 5 × 10^13^ vg/kg (high dose MEA studies), respectively.

### Blood collection

Blood collection was either done through submandibular bleed (survival procedure) or by cardiac puncture following terminal perfusion (at euthanasia) using standard protocols and collected into EDTA-containing tubes. Whole blood was flash frozen.

### Immunosuppression and flow cytometry staining

For immunosuppression, mice were treated with anti-CD20 antibody and with rapamycin. Anti-CD20 was administered twice IV via retro-orbital injection at a dose of 10 mg/kg: one dose 2–3 days prior to viral vector delivery and a second dose 7 days later (4–5 days following the viral vector). Eyes were alternated for IV injections. Rapamycin was administered at a dose of 4 mg/kg, 3 times weekly for 4 weeks following viral vector administration. Rapamycin was added to single-dose peanut butter pellets according to mouse weight, as done previously [23]. B cells in whole blood were quantified using flow cytometry staining. Briefly, whole blood collected on day 30 and at termination (~60–80 days after anti-CD20 administration) was incubated with the following antibody cocktail: CD45-FITC (cat. # 11-0451-82, RRID:AB_465050, *Thermo Fisher Scientific*), CD19-BV421 (cat. # BDB562701, RRID:AB_2737731, *Thermo Fisher Scientific*), B220-APC (cat. #17-0452-82, RRID:AB_469395, *Thermo Fisher Scientific*) and 7AAD (cat. # 50-112-8859, *Thermo Fisher Scientific*). Analysis was done using an LSRII flow cytometer (*BD Biosciences*, Franklin Lakes, NJ, USA). Gating strategy was set to select for singlets, live cells (7AAD (650 nm) negative) >CD45 positive (490 nm) >B220 and CD19 positive (421 nm and 660 nm). Results were analyzed using FACSDiva™ software (*BD Bioscience*).

### Immunohistochemistry

Mice were perfused with PBS, the brains cut along the midline and immersed in 4% PFA for 24 h. Then, brains were cryoprotected (10%–20%–30% sucrose, 24 h each), followed by freezing on dry ice. Brains were then processed and stained for FMRP (cat. # 834702, RRID:AB_2750045, *BioLegend*, San Diego, CA, USA, 1:100) and NeuN (cat. # ab177487, RRID:AB_2532109, *Abcam*, Cambridge, UK, 1:500) at *HistoWiz Inc*. (Brooklyn, NY, USA) following a standard operating procedure and a fully automated workflow. Samples were embedded in OCT followed by frozen sectioning via cryostat at 4 μm. Sequential Tyramide (NEL87100KT, *Akoya Biosciences*, Marlborough, MA, USA) based Immunofluorescence was performed on a Bond Rx Autostainer (*Leica Biosystems*, Nussloch, Germany) with EDTA PH 9.0 Heat Induced Epitope Retrieval (HIER) for 40 min. Whole slide scanning [20×] was performed on an Akoya Polaris IF Scanner with matching OPAL filters o480 and o620.

### DNA and RNA isolation

Tissue from different brain regions (as indicated in the results section) and liver were lysed using the FastPrep-24™ 5G bead beating grinder and lysis system (*MP Biomedicals*, Santa Ana, CA), and DNA and RNA isolated using the KingFisher Apex automated purification system (*Thermo Fisher Scientific*). DNA purity was examined using a Nanodrop spectrophotometer and DNA quantified on the Qubit Fluorometer (*Thermo Fisher Scientific*) using the Qubit dsDNA Broad Range assay kit (*Thermo Fisher Scientific*). The majority (98%) of DNA samples met required purity metrics (260/280 > 1.8, 260/230 > 1.0). Brain RNA concentrations were assessed by Nanodrop and an Agilent 2100 Bioanalyzer system. All samples tested had a 260 nm/280 nm ratio of >1.8 and a RIN score >7, indicating sufficient quality. Liver samples were analyzed using the Qubit RNA IQ kit (*Thermo Fisher Scientific*, RNA IQ value > 8).

### QRT-PCR

Human *FMR1* mRNA was quantified using qRT-PCR. 500 ng of RNA were used for cDNA synthesis using the iScript cDNA Synthesis Kit following manufacturer’s instructions (*BioRad*, Hercules, CA) and quantified by *hFMR1*- and *Gapdh*-specific qRT-PCR using TaqMan™ Fast Advanced Master Mix (*Thermo Fisher Scientific*) and a QuantStudio 7 Real-Time PCR system following manufacturer’s instructions (*Applied Biosystems*, Waltham MA, USA). A standard curve was run with each plate for quality control. PCR efficiency and *R*^2^ values were within accepted ranges for each plate (93–105% and >0.98, respectively). Relative *hFMR1* levels were calculated by normalization to *Gapdh* mRNA levels in each sample (ΔCT method). The following primers and probes were used: hFMR1 forward primer AATCCAAAAGAACAGTGGCATT, hFMR1 reverse primer GGAATCCCAGAAACCTGAACT, hFMR1 probe 5’- FAM/AAGGCTTGGCAGGGTATGGTACC-3’ IWBLFQ/Zen, Gapdh forward primer AATGGTGAAGGTCGGTGTG, Gapdh reverse primer GATGGCAACAATCTCCACTTTG, Gapdh probe 5’- Cy5/ AACGGATTTGGCCGTATTGGGC-3’ IWBLRQ/TAO.

### QPCR

Vector biodistribution was evaluated using a qPCR assay to measure genome copies present within mouse tissue samples. Briefly, the vector copy number (VCN) was determined by TaqMan-based qPCR with 100 ng DNA per reaction using the TaqMan™ Fast Advanced Master Mix and a QuantStudio 7 Real-Time PCR system as described in the manual (*Applied Biosystems*). All assay plates included a six-level standard curve (5E + 06 to 50 copies) run in triplicate, generated from synthesized DNA containing the reference target sequence. Any potential PCR inhibition was evaluated by spiking 300 copies of gBlock into a replicate sample well. For all samples that tested below the lower detection limit, the recovery for this spike was acceptable (81.1–117.9%). The following primers and probes were used: forward primer TCGTGAATGGAGTACCCTGATA, reverse primer CAGCGTATCCACATAGCGTAAA, probe 5’– FAM/CAAGCTTATCGATAATCAACCTCTGGA – 3’ IWBLFQ/ZEN.

### Western blotting

Primary neurons or mouse organs were homogenized in lysis buffer (150 mM NaCl, 1 mM EDTA, 1% Triton x-100, 50 mM Tris HCl (pH = 7.4), 50 mM NaF, 10 mM Na pyrophosphate, and 10 mM Na beta-glycerol phosphate supplemented with cOmplete™, EDTA-free Protease Inhibitor Cocktail and PhosSTOP (*Sigma Aldrich*, St. Louis, MO, USA)). SDS-PAGE and western blot analysis was conducted following standard protocols [24]. Equal amounts of protein were loaded (as determined by Bradford Protein Quantification, between 10 and 30 µg, depending on the organ). Antibodies to human FMRP (cat. # 834601, *BioLegend*, 1:500) were used to detect recombinant FMRP, and antibodies to GAPDH (cat. # NC1573694, *Thermo Fisher Scientific*, 1:2000), βIIITubulin (cat. # 802001, *BioLegend*, 1:2000) and βActin (cat. # A1978, *Sigma Aldrich*, 1:1000) were used as loading controls. Signal was detected with ECL using a digital imager. One sample served as normalization control and was loaded on every gel. Experiments to compare mouse endogenous and human recombinant FMRP expression in the liver were done using a Jess Automated Western Blot System (*Bio-Techne*) using the 12–230 kDa Separation Module (cat. # SM-W004, *Bio-Techne*), EZ Standard Pack 1 (cat. # PS-ST01EZ-8, *Bio-Techne*), the RePlex^TM^ Module (cat. # RP-001, *Bio-Techne*), and Anti-Mouse Detection Module (cat. # DM-002, *Bio-Techne*). 1.92 µg protein per sample were analyzed with antibodies specific to mouse and human FMRP (cat. # 834701, *BioLegend*, 1:10) followed by an anti-mouse GAPDH antibody (cat. # mAbcam 9484, *Abcam*, 1:10). Protein and antibody concentrations were based on optimization experiments to achieve antibody saturation, low background, and antigen-specific signal within the linear range of the assay (*data not shown*).

### Luminex assay to detect FMRP

Tissues for Luminex assays were lysed in mPER buffer (*Thermo Fisher Scientific*), and protein concentration was determined using a Bradford assay. 100 µg of protein was analyzed in quintuplicates or in triplicates using a human FMRP-specific Luminex assay with a standard curve and human recombinant protein as described [7]. For a few samples, insufficient material was recovered, and, therefore, less protein was loaded. The analysis was adjusted accordingly to account for the lesser amount loaded. Five dried blood spots were eluted, combined, and analyzed in triplicate by the Luminex assay as described for human blood [7]. The mean value of the quintuplicates or the triplicates was used as analysis point.

### Behavioral analyses

Order and frequency of behavioral analyses are indicated in the figures for each study. The following behavioral analyses were done but not further discussed due to lack of baseline differences: *nesting behavior*, *social preference assay*, and *Morris water maze*. Description of these tests is provided in the Supplementary Information.

### Audiogenic seizures

Audiogenic seizures (AGS) at postnatal day 21 were tested at CCHMC as described [25]. Briefly, on the day of AGS testing, mice were placed in pairs into a new cage, acclimated for 30 min, and exposed to a 2-min 120 dB sound, followed by 1 min of silence and then a second 2-min sound. Response of the mice to the audiogenic seizure was scored on a scale of 0–4 as follows: 0 = no change; 1 = wild running; 2 = clonic seizure; 3 = tonic seizure; 4 = death. The highest score within either of the sound exposures was reported. Following AGS, mice were housed in groups of up to four separated by sex. AGS at postnatal day 63 were tested at *Melior Discovery, Inc*. Briefly, one mouse at a time was placed in a clear cylindrical Plexiglas chamber with an alarm mounted at the top. Sound exposure and scoring were described above. These mice were euthanized immediately after AGS.

### Nest removal/digging behavior

Two to three cages were placed under the hood equal distance apart. The cage lid, water bottle, and wire food top were removed from the cages to ensure visibility. Mice habituated for 10 min. After habituation, digging behavior was video recorded for 10 min with the nest. Then, the nest was removed, and digging was recorded for another 10 minutes without the nest. Findings are reported as time spent digging.

### Open field activity

Mice were placed in a 41 × 41 cm^2^ activity chamber for 1 hour. Activity, measured by photobeam breaks, was assessed every 5 min, including time spent in the center [26].

### MEA surgery

Surgeries to implant skull surface multielectrode arrays (MEA, *NeuroNexus Technologies*, Ann Arbor, MI, USA) were performed 4 weeks after vector or vehicle administration as described [27]. Briefly, prior to implantation the probe ribbon was protected with a silicone layer and, together with the reference wires, secured with electric tape. On the day of surgery, mice were anesthetized, and the skull was exposed with a single sagittal incision through the skin. Three 1-mm holes for screw placement were drilled in the skull, and the MEA was placed on the skull (aligned to bregma). The MEA ribbon and probe connector were positioned vertically using two wooden sticks to hold them in place and facilitate connecting to the commutator. Saran wrap and Teflon were used to protect the MEA, the skin was sutured and then covered with dental cement. Triple antibiotic ointment to avoid infections was applied around the edges of the dental cement. Mice received carprofen before and after surgery for pain management. All mice were single-housed after surgery to avoid chewing off the head mount.

### MEA recording and analysis

One week after MEA surgery, mice were lightly anesthetized with isoflurane to connect the MEA with the head stage and commutator. Mice were then placed in the recording chamber for 15 min of habituation, followed by 30 min of recording at a 1000 Hz sampling rate. Afterward, mice were disconnected from the commutator and placed back in the home cage. Spectral power was calculated from murine resting EEG data. The primary outcome for this study was absolute low gamma (30–59 Hz) power. In preprocessing, the data, anonymized via coded filenames to mask group and individual identities, were exported to EEGLAB (MATLAB 2018a, *The MathWorks Inc*., Natick, MA, USA). The EEG was down sampled to 500 Hz, high-pass filtered at 0.5 Hz, and notch filtered between 57 and 65 Hz to remove electrical line noise. Visual inspections for artifacts were conducted by trained personnel, interpolating over bad channels using spherical spline interpolation, not exceeding 10% of data per subject. The data were then average referenced to enhance signal clarity. A standard Welch method was employed from the eegUtils R package (default parameters) to estimate the Power Spectral Density (PSD) of continuous MEA recordings. Eighty [80] epochs from each dataset were randomly selected, ensuring stationary signal conditions, and applied the Welch method to calculate the PSD for each channel. The resulting PSD estimates were used to investigate the gamma band power distribution across the MEA channels, providing insights into the spectral characteristics of high-frequency brain activity associated with the FXS mouse model. Specifically, the results focused on the gamma frequency band ranging from 30 to 55 Hz.

### Statistical analyses

Statistical analyses were done using GraphPad Prism v10.5.0 (*GraphPad*, San Diego, CA, USA), SPSS (*IBM*, Armonk, NY, USA) or R software. Data sets were analyzed for normality using the Shapiro-Wilk test and for equal variance using Bartlett’s test (for data analyzed by one-way ANOVA). Parametric or nonparametric statistical tests were performed as appropriate. Multifactorial designs (ANOVA, repeated measures (RM)-ANOVA, mixed-effect models) were used as determined by the experimental design and followed with post hoc tests accounting for multiple testing. All RM ANOVA assumed sphericity, whereas mixed effect models were analyzed using Greenhouse-Geisser correction to avoid type 1 errors. Depending on the assay and the research question, we used different post hoc tests to correct for multiple testing: Dunn’s and Dunnett’s post hoc tests were used when comparing to one control sample for nonparametric and parametric data, respectively, and Tukey’s tests were used when comparing simple effects within rows for RM-ANOVA to compare vectors within timepoints or organs. Fisher’s exact tests for audiogenic seizure analysis were not corrected for multiple testing, as these were exploratory. All pairwise comparisons were two-sided. Target sample sizes were pre-determined in study protocols for each experiment and based on goal and scope of the experiment (e.g., qualitative/pilot: initial expression and biodistribution studies, versus quantitative/efficacy: audiogenic seizures, behavior and MEA) and expected effect sizes based on the literature. They were adjusted based on litter sizes (audiogenic seizures in mice injected as neonates: targeted 10, actual 10–16) or available genotypes and/or early deaths (audiogenic seizures in mice injected at 5 weeks of age: targeted 12, actual 11–12; behavioral analyses: targeted 10, actual 8–14) or usable data (MEA analysis, targeted 10, actual 7–9 due to unusable MEA signal). The number of different litters was 13 for audiogenic seizure experiments in mice injected as neonates and 39 for behavioral experiments. The number of litters used in experiments in which mice were directly purchased from The Jackson Laboratories is unknown, but based on group sizes, delivery dates, expected litter sizes and sex distribution, we estimate that they came from at least three different litters for each experiment. Sample sizes and statistical tests are stated in the text as well as in Supplementary Tables. Bar graphs and error bars represent mean ± standard error of the mean, and dots are individual mice. Mice were excluded from analysis if immunohistochemical or biochemical analyses revealed “missed target,” i.e., did not show FMRP expression. Data were screened for outliers using the ROUT method (Robust Regression and Outlier Removal*, GraphPad*). Two outliers were identified and removed for the digging/nest removal assays (one each in the WT Vehicle groups). Dosing groups were randomized and evenly distributed between cohorts/litters, and analyzers were blinded to treatment. Except for the initial biodistribution study and comparison of FMRP expression in the liver, only male mice were used in this study.

## Results

### An AAV/CAG gene therapy vector is expressed in hippocampal and cortical neurons of the mouse brain

**Fig. 1:**
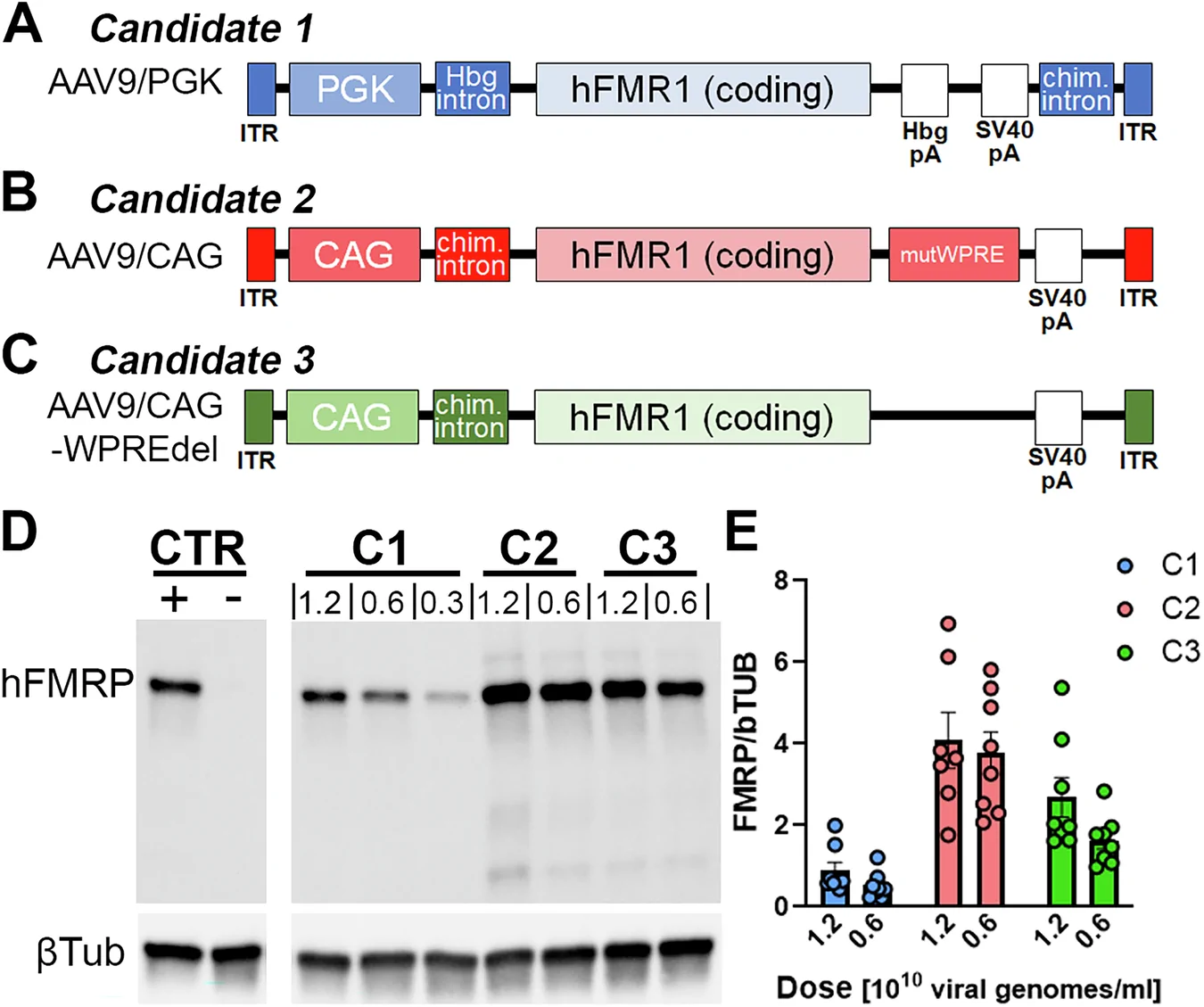
Candidate hFMR1 viral vectors express human FMRP in a dose-dependent manner in primary cortical mouse cultures. **A**–**C** Schematics of the three viral vector candidates tested in expression and audiogenic seizure experiments (**A** *Candidate 1 (C2)*, FMRP expression driven by the phosphoglycerate kinase (PGK) promoter, **B** *Candidate 2 (C2)*, FMRP expression driven by a chimeric chicken βActin/cytomegalovirus (CAG) promoter, **C** *Candidate 3 (C3)*, as C2 with a deletion of the woodchuck hepatitis virus posttranscriptional regulatory element (WPRE)). **D** Qualitative western blot analysis of human FMRP expression in *Fmr1* KO mouse primary cultures transduced at 7 days in vitro and collected 7 days later. Numbers on top of blots indicate concentration of viral vector genomes in 10^10^ viral genomes/ml. Positive control is lysate from neurons transduced with a different viral preparation generated at CCHMC previously shown to express human FMRP, negative control is untransduced neuronal lysate. **E** Semiquantitative analysis of western blots showed a significant effect of virus with C2 leading to the highest FMRP expression (2-way ANOVA, p(interaction) = 0.6; p(dose) = 0.09, *p(virus)<0.0001, *n *= 8 for C1(1.2), C1(0.6), C(0.6), C3(1.2), and C3(0.6); *n* = 7 for C2(1.2). *N* are independent transductions of four separate embryonic culture preparations, bar diagrams are means ± SEM.

**Fig. 2:**
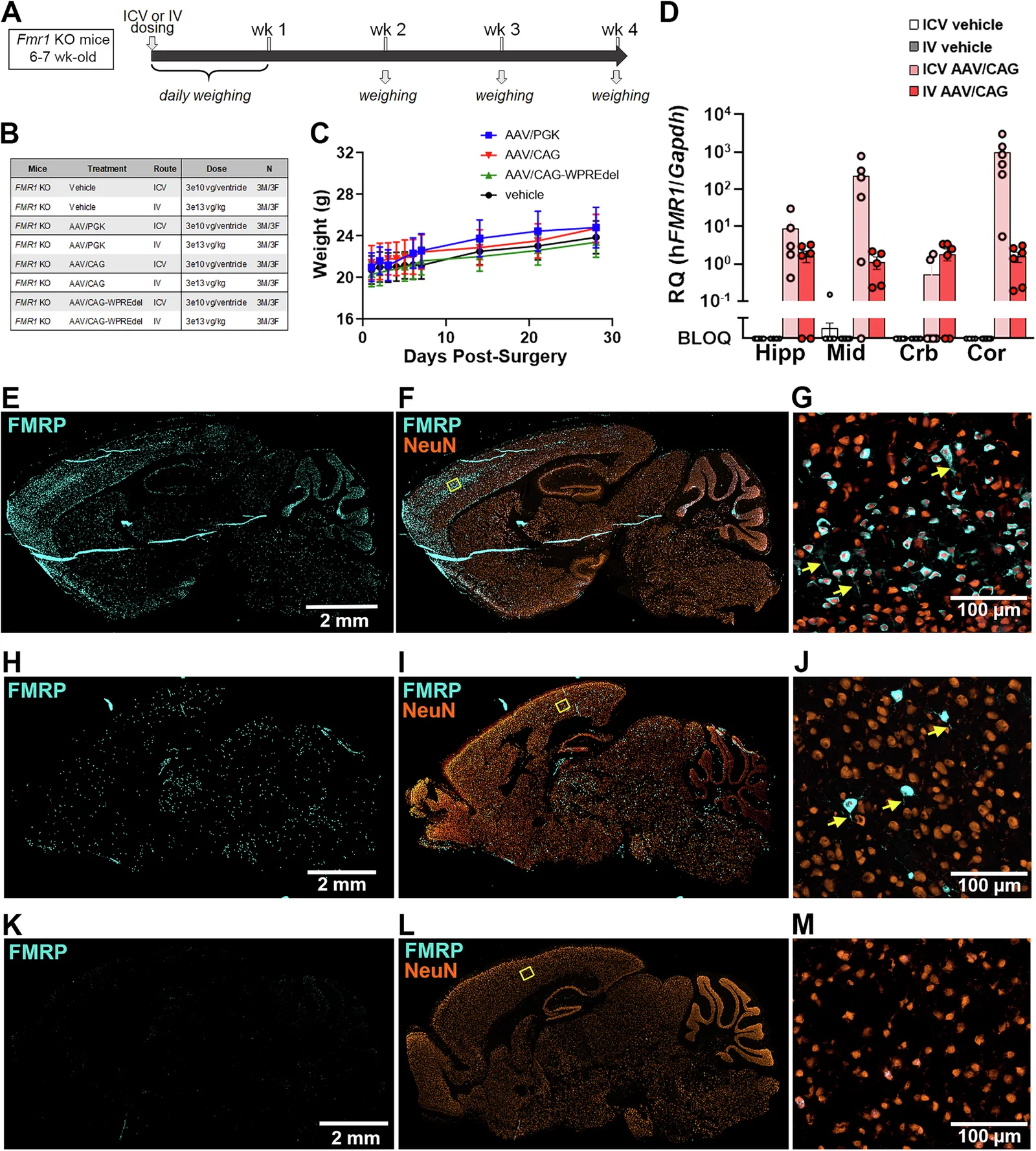
Intravenous and intracerebroventricular injection of AAV/CAG is safe in male and female *Fmr1* KO mice and leads to widespread FMRP expression in the brain. **A** Timeline of experiments. **B** Dosing groups including sample sizes (*M* = male, *F* = female). **C** Mice gained weight as expected, regardless of viral vector and dosing route (mixed-effects model, p(day)<0.0001, p(viral vector) = 0.923). **D** QRT-PCR analysis for virally expressed *hFMR1* mRNA after IV or ICV dosing of AAV/CAG (*C2 in* Fig. 1) shows expression in hippocampus, cortex, cerebellum and midbrain (mixed-effects model, p(brain region) = 0.079, p(vector) = 0.007, p(interaction) = 0.015, no pairwise comparisons significant, sample sizes between 4 and 6 depending on brain region and viral vector). Bar graphs and error bars represent mean with standard errors. **E**–**M** Immunohistochemical staining of sagittal brain slices shows widespread FMRP expression across the brain after ICV (**E**–**G**) and IV (**H**–**J**) dosing, including the characteristic somatodendritic expression in neurons of the cortex (**G**, **J**) whereas background staining in mice ICV injected with vehicle showed only background signal (**K**–**M**). Neurons were identified with NeuN staining. Yellow squares in (**F**, **I** and **L**) indicate approximate areas shown in magnifications **G**, **J** and **M**. Yellow arrows in (**G**) and (**J**) point to dendrites. Western blot results are shown in Supplementary Fig. 1 and Supplementary Table 1. Hipp hippocampus, Mid midbrain, Crb cerebellum, Cor cortex.

Three different AAV9 viral vectors for strong and constitutive expression of the coding sequence of human *FMR1* (*hFMR1*) isoform 1 in mammalian cells were generated: AAV/CAG, AAV/CAG-WPRE*del* and AAV/PGK (Fig. 1A–C). Expression of transgene (*hFMR1*) encoded by the viral vectors was tested in vitro in cultured primary mouse cortical neurons and in vivo in adolescent mice post-transduction. AAV/PGK expresses the transgene under the control of the human phosphoglycerate kinase 1 promoter (Fig. 1A, Candidate 1). AAV/CAG expresses the transgene under the control of the strong constitutive hybrid promoter of CMV and chicken β-actin (CAG) (Fig. 1B, Candidate 2). Candidate 2 contains a mutated woodchuck hepatitis virus posttranscriptional regulatory element (WPRE). The mutated WPRE strongly reduces the oncogenic potential of the original WPRE while retaining its beneficial effects on expression and mRNA stability [28]. To test if the WPRE was needed for good expression and efficacy in vivo, it was removed in the third viral vector, AAV/CAG-WPREdel (Fig. 1C, Candidate 3). Viral vector constructs were confirmed by sequencing (*data not shown*) and used to generate viral particles of AAV9 serotype. Mouse embryonic cortical neurons were transduced with viral vectors at 7 days in vitro (DIV), followed by collection and western blot analysis 7 days later. Dose-dependent FMRP expression with each viral vector was demonstrated, with the strongest expression coming from AAV/CAG-hFMR1 and the lowest expression with AAV9-PGK (Fig. 1D). Semiquantitative western blot analysis confirmed the qualitative impression (Figs. 1E, 2-way ANOVA, *p*(interaction) = 0.6; *p*(dose) = 0.09, *p*(virus) < 0.0001).

We next tested transgene expression in vivo with all three candidate viral vectors four weeks after ICV or IV administration in adolescent male and female *Fmr1* knockout mice (timeline and dosing groups shown in Fig. 2A, B). Mice tolerated the viral vectors well and showed expected weight gain after all treatments (Fig. 2C, mixed-effects model, p(day)<0.0001, p(vector)=0.923). No viral vector-related deaths or adverse health events were observed. Quantitative real-time PCR (qRT-PCR) analysis of brain tissue of AAV/CAG-hFMR1 injected mice showed strong expression of h*FMR1* mRNA in hippocampus, midbrain, cerebellum, and cortex. As expected, expression was higher in ICV- compared with IV-injected mice in all brain regions except for cerebellum (Fig. 2D, mixed-effects model, p(brain region)=0.079, p(vector)=0.007, p(interaction)=0.015, no pairwise comparisons significant). FMRP-specific immunohistochemistry (IHC) showed that ICV injection of AAV/CAG-hFMR1 led to FMRP expression mainly in prefrontal areas of the brain (Fig. 2E, F), whereas IV injection led to broader expression across the entire brain with slightly higher expression in the midbrain (Fig. 2H, I). On a cellular level, FMRP expression showed the typical somatodendritic distribution of endogenous FMRP in neurons (Fig. 2G, J). ICV injection with vehicle only showed background staining (Fig. 2K–M). Brain biodistribution analyses were focused on AAV/CAG due to its better efficacy to rescue audiogenic seizures in adolescent mice (see below). Western blot analysis of several organs apart from the brain from mice injected with either of the three tested viral vectors showed consistent expression of FMRP in liver and heart of IV injected mice, with light expression in the liver of some ICV-injected mice, independent of the viral vector administration (Supplementary Fig. 1). No clear FMRP-positive signal on western blots was detected in kidney, ovaries, DRG, muscle or lung (Supplementary Table 1), consistent with the expected biodistribution of the AAV9 serotype.

### Administration of AAV9-hFMR1 in neonate *Fmr1* KO mice reduces susceptibility to audiogenic seizures

**Fig. 3:**
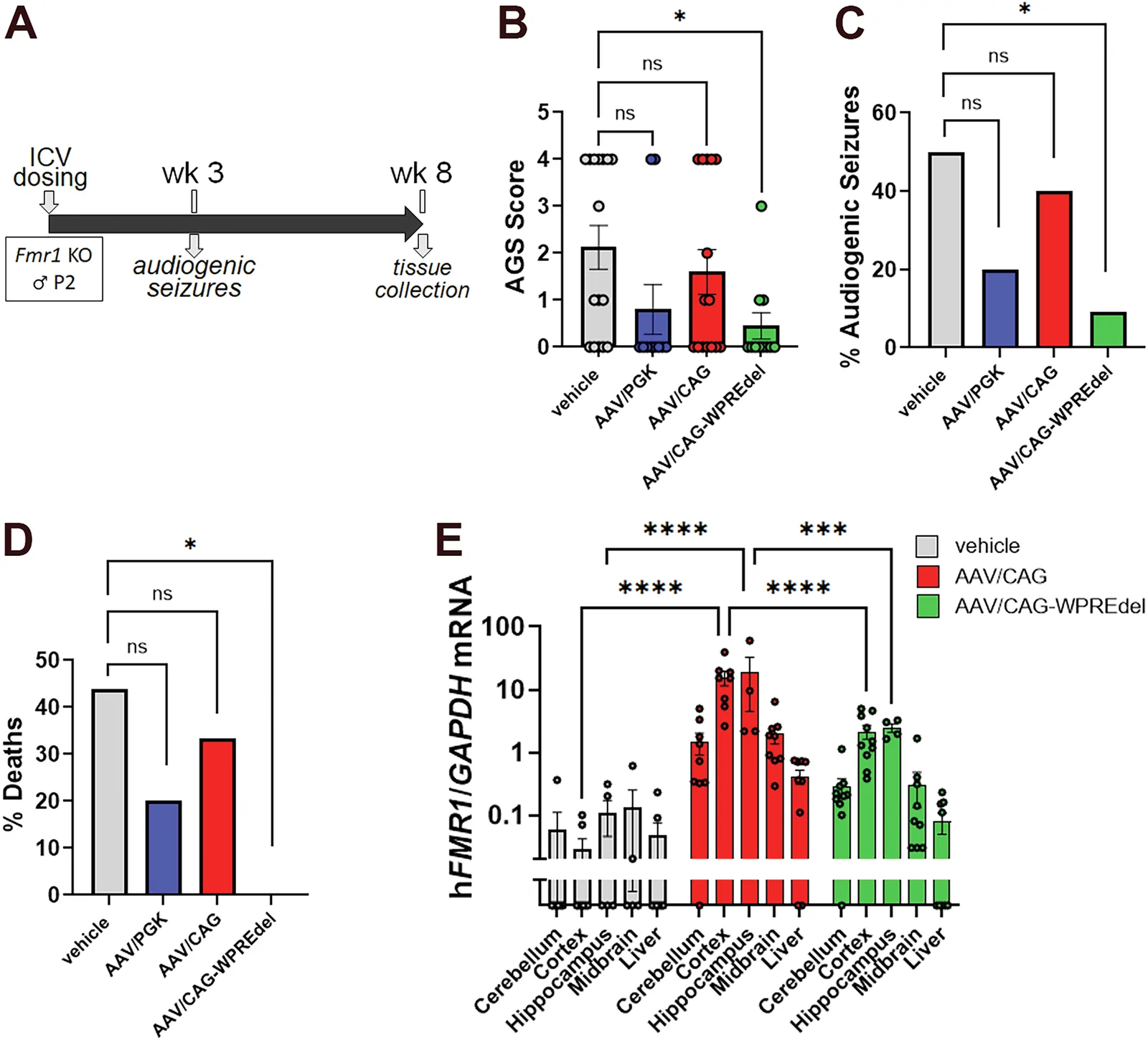
Neonatal administration of hFMR1-expressing AAV reduces susceptibility to audiogenic seizures in male *Fmr1* KO mice at three weeks of age. **A** Timeline of experiments. Viral vectors were delivered ICV. **B** Audiogenic seizure scores are overall reduced after AAV/hFMR1 dosing, with significantly reduced seizure score after dosing with AAV/CAG-WPREdel (Kruskal Wallis test with Dunn’s post hoc test, H = 8.1, p = 0.044, *p = 0.047, n(vehicle) = 16, n(AAV/PGK) = 10, *n*(AAV/CAG) = 15, n(AAV/CAG-WPREdel)=11). **C** Seizure probability is overall reduced after dosing with AAV/hFMR1, with significantly reduced occurrence of seizure after dosing with AAV/CAG-WPREdel (Fisher’s exact tests, *p = 0.042, n as in **B**). **D** Number of deaths is overall reduced after dosing with AAV/hFMR1, with significantly reduced deaths after dosing with AAV/CAG-WPREdel (Fisher’s exact tests, *p = 0.022, n as in **B**). **E** *hFMR1* mRNA is increased over background in brains of 8-week-old mice that received neonatal ICV injection of AAV/CAG or AAV/CAG-WPREdel. Deletion of WPRE reduces *hFMR1* expression by roughly 7-fold across all brain regions and the liver. Consistent with ICV injections, *hFMR1* levels in cortex and hippocampus were highest (two-way ANOVA with Tukey’s post hoc test, *p*(organ) = 0.0002,* p*(viral vector) <0.0001, p(interaction)=0.0002, ***p < 0.0005, ****p < 0.0001, sample sizes between 4 and 10). Comparison of mice with and without visible leak during injection shown in Supplementary Fig. 2. Bar graphs and error bars represent mean with standard errors. Statistics and sample sizes are shown in Supplementary Table 2.

We next tested the three viral vectors for their efficacy to rescue a core phenotype in *Fmr1* KO mice, susceptibility to audiogenic seizures [29]. In the first study, neonatal mice were injected ICV bilaterally with one of the three viral vector candidates or vehicle followed by test for audiogenic seizure susceptibility three weeks later (6 × 10^9^ vg/ventricle, *n*(vehicle) = 16, *n*(AAV/PGK) = 10, *n*(AAV/CAG) = 15, *n*(AAV/CAG-WPREdel)=11, Fig. 3A). On average, all three vectors reduced audiogenic seizure scores (Fig. 3B, Kruskal Wallis test, *p* = 0.044, Dunn’s post hoc tests, *p = 0.047), percent seizures (Fig. 3C, Fisher’s exact tests, *p = 0.042), and percent deaths (Fig. 3D, Fisher’s exact tests, *p = 0.022) in male *Fmr1* KO mice compared with WT mice. When mice with minor leaks of viral vector during injection were removed from the analysis, results remained the same (Supplementary Fig. 2A-C). QRT-PCR analysis showed differential expression depending on viral vector and brain region/organ, with high expression of *hFMR1* mRNA in cortex and hippocampus ~8 weeks after administration of AAV/CAG-hFMR1 and AAV/CAG-WPREdel-hFMR1, whereas levels in cerebellum, midbrain and liver were lower (Figs. 3E, 2-way ANOVA, p(interaction) = 0.0002, *p*(tissue type) = 0.0002, *p*(viral vector) < 0.0001, sample sizes between 4 and 10 depending on brain region and viral vector, for details see Supplementary Table 2). As expected, the viral vector containing the mutWPRE yielded significantly higher expression levels. Each group contained 1–2 samples in which a unilateral leak was observed during viral vector application; however, exclusion of these samples did not change the results (Supplementary Fig. 2D).

### Administration of AAV9-hFMR1 in adolescent *Fmr1* KO mice reduces susceptibility to audiogenic seizures

**Fig. 4:**
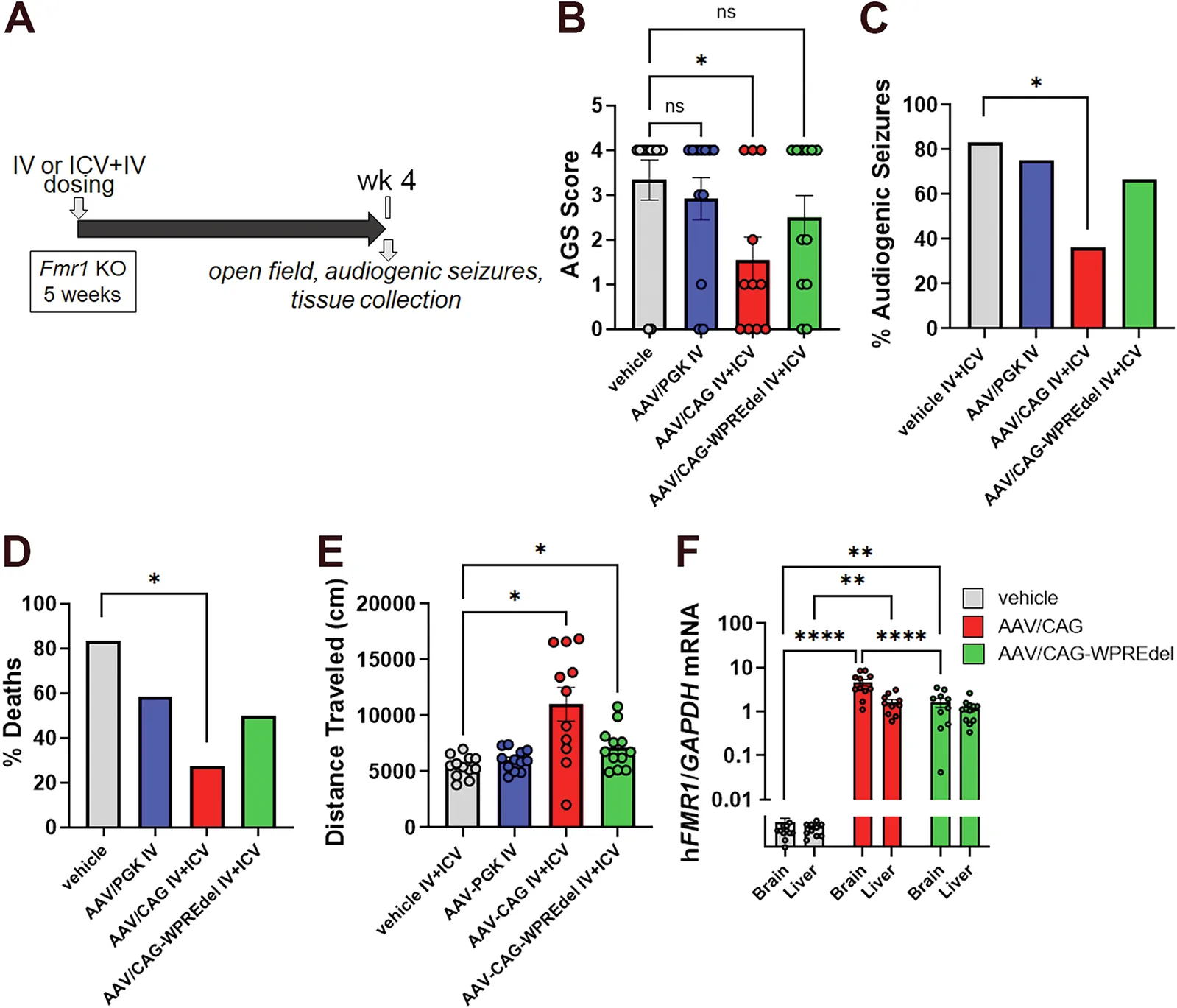
Administration of hFMR1-expressing AAV in adolescent male *Fmr1* KO mice reduces susceptibility to audiogenic seizures after 4 weeks. **A** Timeline of experiments. AAV/PGK was delivered IV (3 × 10^13^/kg), whereas AAV/CAG and AAV/CAG-WPREdel were delivered via a dual IV/ICV route (3 × 10^13^vg/kg and 3 × 10^10^vg/ventricle). **B** Audiogenic seizure scores are significantly reduced after dosing with AAV/CAG administered IV/ICV (Kruskal Wallis test with Dunn’s post hoc test, *H* = 6.5, *p* = 0.09, *p = 0.038, n(vehicle)=12, *n*(AAV/PGK) = 12, *n*(AAV/CAG) = 11, *n*(AAV/CAG-WPREdel) = 12). **C** Seizure probability is significantly reduced after dosing with AAV/CAG administered IV/ICV (Fisher’s exact tests, *p = 0.036, *n* as in **B**). **D** Number of deaths is significantly reduced after dosing with AAV/CAG (Fisher’s exact tests, *p = 0.012, n as in **B**). **E** Open field activity is significantly increased in *Fmr1* KO mice after administration of AAV/CAG and AAV/CAG-WPREdel vector (Welch’s ANOVA with Dunnett’s T3 post hoc test, *p(veh-CAG) = 0.011, *p(veh-CAG-WPREdel) = 0.042, n as in **B**). **F** *hFMR1* mRNA is increased over background in brains and livers of *Fmr1* KO mice 4 weeks after dual IV/ICV administration of AAV/CAG or AAV/CAG-WPREdel. (two-way ANOVA with Tukey’s post hoc test, p(organ) = 0.0003, p (vector) < 0.0001, p(interaction) = 0.0002, **p = 0.009, ****p < 0.0001, brain:, n(vehicle) =12, n(AAV/CAG) = 11, n(AAV/CAG-WPREdel) = 10, liver: n (vehicle) =11, *n*(AAV/CAG) = 10, *n*(AAV/CAG-WPREdel) = 12; two data points within y axis gap). Bar graphs and error bars represent mean with standard errors. Detailed statistics are shown in Supplementary Table 2.

Postnatal injections mimic *in utero* administration in humans, which is currently not a viable approach for clinical trials in FXS. Therefore, we next conducted a similar study in adolescent mice. Here, *Fmr1* KO mice in FVB/J background were injected at 5 weeks of age and tested for locomotor activity and audiogenic seizures at 9 weeks (Fig. 4A). FVB/J background was chosen based on previous studies showing increased audiogenic seizure susceptibility of *Fmr1* KO mice in this background at older ages [30]. The three viral vector candidates were injected using a dual ICV/IV route to achieve broad viral vector distribution in the brain for this initial efficacy test. Because we had originally hypothesized that AAV/PGK-hFMR1 would be the optimal viral vector due to its moderate but ubiquitous expression levels and good safety profile (mainly based on work using lentivirus [31, 32]), we added a group, in which AAV/PGK-hFMR1 was dosed IV only. Dual ICV/IV injection of AAV/PGK-hFMR1 led to death within 1-2 days of surgery. It is unclear if these deaths were related to the viral vector because of the short timeline to death after dosing and because ICV injections of similar amounts of AAV/PGK-hFMR1 in 6-week-old C57BL/6J mice did not lead to mortality (*data not shown*). Notably, only AAV/CAG-hFMR1 administered ICV/IV reduced audiogenic seizure scores (Fig. 4B, 1-way ANOVA, *p* = 0.073; Dunnett’s multiple comparison test *p < 0.05), the incidence of seizures (Fig. 4C, Fisher’s exact tests, *p < 0.05), and deaths (Fig. 4D, Fisher’s exact tests, *p < 0.05). Both AAV/CAG-hFMR1 and AAV/CAG-hFMR1-WPREdel treatment increased locomotor activity (Fig. 4E, Welch’s ANOVA, *p* = 0.0036 with Dunnett’s T3 multiple comparison test, *p < 0.05); however, due to the inconsistent phenotypes in the open field assay of *Fmr1* KO mice, it is unclear how this result relates to the *Fmr1* KO phenotype [33, 34]. QRT-PCR analysis showed higher *hFMR1* mRNA expression levels in the brain 4 weeks after administration of AAV/CAG-hFMR1 than AAV/CAG-WPREdel-hFMR1 with comparable levels in the liver (Figs. 4F, 2-way ANOVA, *p*(interaction) = 0.0002, *p*(tissue type) = 0.0003, *p*(viral vector) < 0.0001, Tukey’s multiple comparisons test, **p < 0.01; ****p < 0.0001). Sample sizes and statistics are provided in Supplementary Table 2.

### AAV/CAG-hFMR1 improves digging behavior without altering overall activity levels

**Fig. 5:**
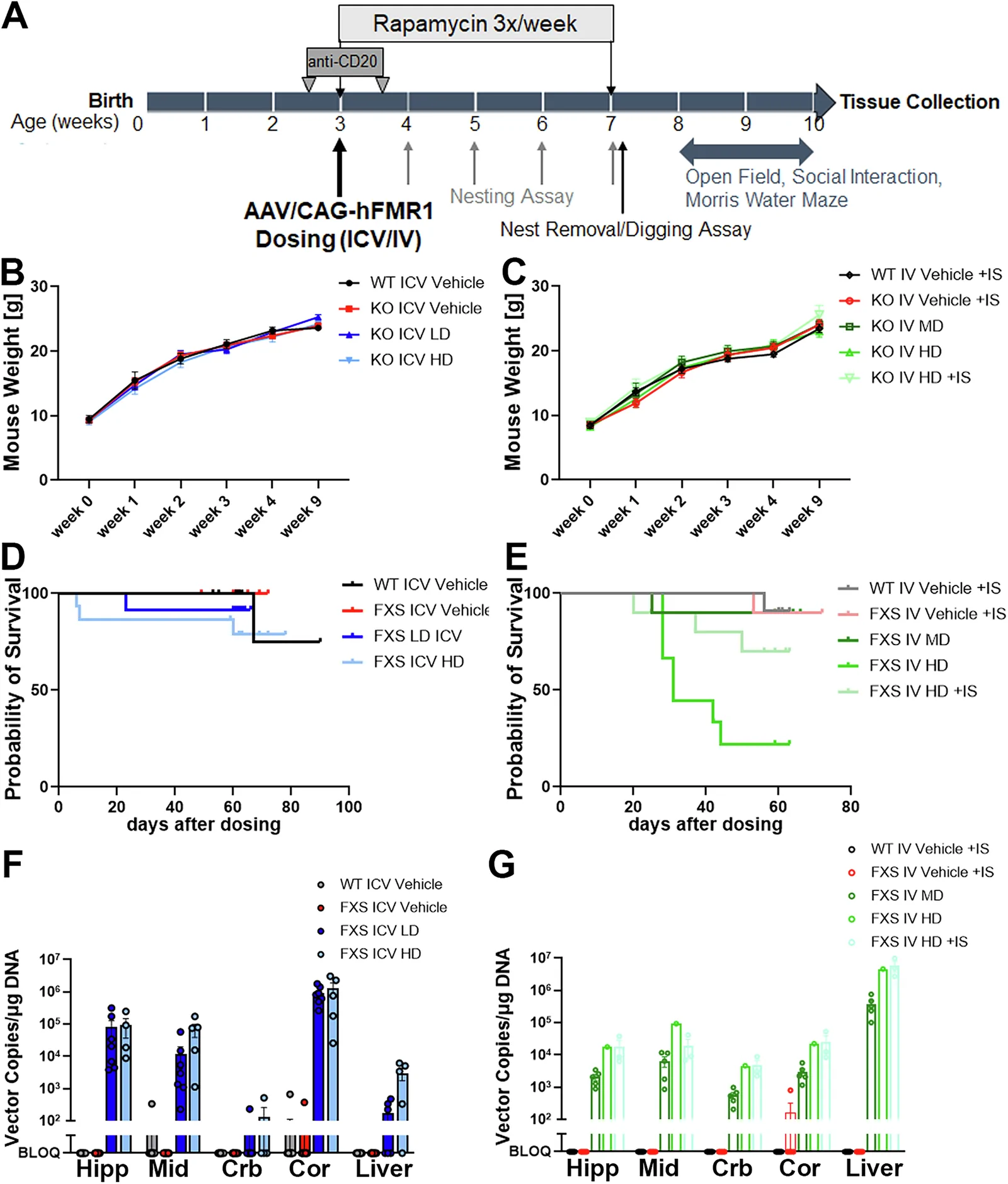
ICV or IV dosing of AAV/CAG-hFMR1 is overall well tolerated and leads to dose-dependent viral vector transduction. **A** Timeline including immunosuppression and dosing. Dosing and delivery routes are shown in Table 1. **B**, **C** All mice gained weight as expected over 9 weeks after dosing, with no significant effects of vector, dose, route or immunosuppression (**B** ICV dosing groups, mixed effects model, *p* (week)<0.0001, *p* (group) = 0.73, *p* (interaction) = 0.45, *n* (wt veh) = 14, *n* (KO veh) = 13, *n* (KO LD) = 11, *n* (KO HD) = 13; **C** IV dosing groups, mixed effects model, *p* (week)<0.0001, *p* (group) = 0.76, *p*(interaction) = 0.71, *n*(wt veh IS) = 11, *n*(KO veh IS) = 10, *n*(KO MD) = 10, *n*(KO HD) = 8, *n*(KO HD IS) = 9). Viral vector administration did not lead to increased mortality except for mice that received high dose of viral vector IV without immunosuppression (**D** ICV dosing groups, Log rank (Mantel-Cox) test, *Χ*^2^(3) = 2.72, *p* = 0.436, **E** IV dosing groups, Log rank (Mantel-Cox) test, *Χ*^2^(4) = 23.14, *p* = 0.0001, sample sizes as in **B**, **C**). Bar graphs and error bars represent mean with standard errors. **F** Viral vector genome is detected in hippocampus, cortex, midbrain and at lower levels in cerebellum and liver after ICV dosing. The high dose overall leads to increased vector copy number (mixed effects model, *p*(organ)<0.0001, *p*(group)=0.0001, *p*(interaction) = 0.0002, *n*(WT veh) = 12, *n*(KO veh) = 9, *n*(KO LD) = 7, *n*(KO HD) = 5). **G** Viral vector genome is detected at similar levels in hippocampus, cortex, cerebellum, and midbrain and at higher levels in liver after IV dosing. High doses led to increased viral vector copies compared with mid dose (mixed effects model, *p*(organ) < 0.0001, p(group) <0.0001, *p*(interaction) < 0.0002, *n*(WT veh IS) = 7, *n*(KO veh IS) = 5, *n*(KO MD) = 5, *n*(KO HD) = 1, n(KO HD IS) = 3). Bar graphs and error bars represent mean with standard errors. Detailed statistics are shown in Supplementary Table 3. *IS* immunosuppression, *Hipp* hippocampus, *Mid* midbrain, *Crb* cerebellum, *Cor* cortex. Detailed statistics are shown in Supplementary Table 3. Successful B-cell depletion is shown in Supplementary Fig. 3.

Because of the promising results in the audiogenic seizure experiments in adolescent mice, we focused further efficacy studies on vector AAV/CAG-hFMR1. We first tested the potential of the *FMR1* gene therapy to rescue behavioral deficits in *Fmr1* KO mice. Littermate male *Fmr1* KO and WT mice were generated by breeding female *Fmr1* heterozygous mice with male WT mice. Mice were dosed between postnatal day 21 and 24 and the viral vector was administered ICV or IV in high and low doses each. A list of all groups and vector doses is shown in Table 1. Immunosuppression during and after AAV vector administration has been shown to increase transduction efficiency and can prevent potential immune responses against the introduced capsid and transgene [35]. Therefore, to increase transduction efficiency and improve safety, we included an immunosuppressant treatment before and after the viral vector administration for mice receiving a high dose IV. In these mice, anti-CD20 treatment to ablate B cells was administered 1–3 days before and 1–3 days after viral vector administration, followed by a 4-week 3 times/week rapamycin regimen. This treatment led to significant depletion of B cells after 4 weeks, which were replenished after 8 weeks (Supplementary Fig. 3). To ensure that immunosuppression did not affect the baseline phenotype, the vehicle-injected WT and *Fmr1* KO groups received the same anti-CD20/rapamycin treatment as the high-dose group. Mice were weighed and tested for nesting behavior weekly for four weeks starting 1 week after dosing. Four to five weeks after dosing, mice were tested in a battery of behavioral assays, namely nest removal/digging, open field activity, social preference, and Morris water maze (in order from least to most stressful). After completion of the behavioral assays, nine to eleven weeks after dosing, mice were euthanized, and major organs collected (brain, liver, lung, heart, spinal cord, kidney, gonads, calf muscle, dorsal root ganglia; only brain analyzed in this study). A timeline is shown in Fig. 5A. ICV and IV groups were analyzed separately. Mice in all groups gained weight as expected with no significant effects of vector (Fig. 5B, ICV group, mixed-effects model, p(week) < 0.0001, *p*(vector)=0.735, *p*(interaction)=0.452; Fig. 5C, IV group, mixed-effects model, *p*(week) < 0.0001, p(vector) = 0.758, *p*(interaction) = 0.706). Mice tolerated immunosuppression well, with no negative effects on weight gain or survival (Fig. 5D, E). We noted increased mortality 4–6 weeks after IV administration of high doses of viral vector without immunosuppression (IV HD, 1 × 10^14^ vg/kg). AAV9 has strong tropism for liver, in addition to the brain, and thus IV dosing is expected to lead to accumulation of FMRP in the liver. To assess potential liver toxicity caused by excess FMRP as a cause of mortality in mice that were IV administered with high dose of AAV/CAG-hFMR1 we compared FMRP expression in the liver of mice injected with 3 × 10^13^ vg/kg AAV/CAG-hFMR1 (as in Fig. 2, mid dose between the low and high doses in Fig. 4) with endogenous FMRP expression in the liver of wild type mice. Western blot analyses showed that recombinant FMRP levels after AAV/CAG-hFMR1 IV dosing in *Fmr1* KO mice were variable and, on average 3.8× higher than endogenous FMRP levels in untreated wild type mice (Supplementary Fig. 4). However, histopathological examinations showed only mild hepatocellular degeneration in only one out of three mice that were IV administered with high dose AAV/CAG-hFMR1 (data not shown). AAV9 also shows tropism for heart, but likewise, no prominent histopathology in the heart was detected. Administration of the same high dose of AAV in immunosuppressed mice (IV HD + IS) significantly increased survival, suggesting that deaths might have been caused by an immune response to the viral vector and were not due to liver or cardiac toxicity caused by FMRP overexpression (Fig. 5D, Log rank (Mantel-Cox) test: *p* = 0.44; Fig. 5E, Log rank (Mantel-Cox) test: *p* = 0.0001). Successful dosing for all groups was confirmed with qPCR quantifying viral vector copies at the conclusion of the study. As expected, higher doses showed higher levels of viral vector overall in hippocampus, midbrain, cerebellum, cortex, and liver regardless of dosing route (Fig. 5F, G). Viral vector copy number in hippocampus, midbrain and cortex was higher in ICV-dosed mice, whereas copy number in the liver was higher in IV-dosed mice, confirming the expected dosing route-dependent biodistribution.

**Table 1:** Groups and sample sizes of mice that were enrolled and tested for dose finding/efficacy.

Mouse Model	Treatment Group	Route	Dose	Dose Volume	IS	Mice Enrolled	Mice Analyzed
C57Bl/6	Vehicle	ICV	N/A	1.3 µl/ventricle	**-**	18 M	14 M
FMR1 KO	Vehicle	ICV	N/A	1.3 µl/ventricle	**-**	16 M	13 M
FMR1 KO	Low Dose	ICV	2E10 vg/g est. brain mass	1.3 µl/ventricle	**-**	15 M	11 M
FMR1 KO	High Dose	ICV	1E11 vg/g est. brain mass	1.3 µl/ventricle	**-**	19 M	13 M
C57Bl/6	Vehicle	IV	N/A	6.8 ml/kg	**+**	11 M	11 M
FMR1 KO	Vehicle	IV	N/A	6.8 ml/kg	**+**	11 M	10 M
FMR1 KO	Mid Dose	IV	2.5E13 vg/kg	6.8 ml/kg	**-**	11 M	9 M
FMR1 KO	High Dose	IV	1E14 vg/kg	5.9 ml/kg	**-**	10 M	2 M
FMR1 KO	High Dose	IV	1E14 vg/kg	5.9 ml/kg	**+**	11 M	7 M

Brain weight for a P21 mouse was estimated as 0.39 g [22]. *M* indicates male mice. In most groups, some mice were not analyzed in all assays due to missed doses or early death.

We did not detect baseline differences between vehicle-treated *Fmr1* KO and WT littermates for nesting, social preference and Morris water maze assays and no significant effects of treatment (Supplementary Figs. 5–8). Because of the lack of a baseline effect, the interpretation of these assays is difficult, and thus, these data are not further discussed. Two behavioral assays showed baseline deficits in *Fmr1* KO versus WT mice, namely the nest removal/digging assay and open field activity, which are described below.

**Fig. 6:**
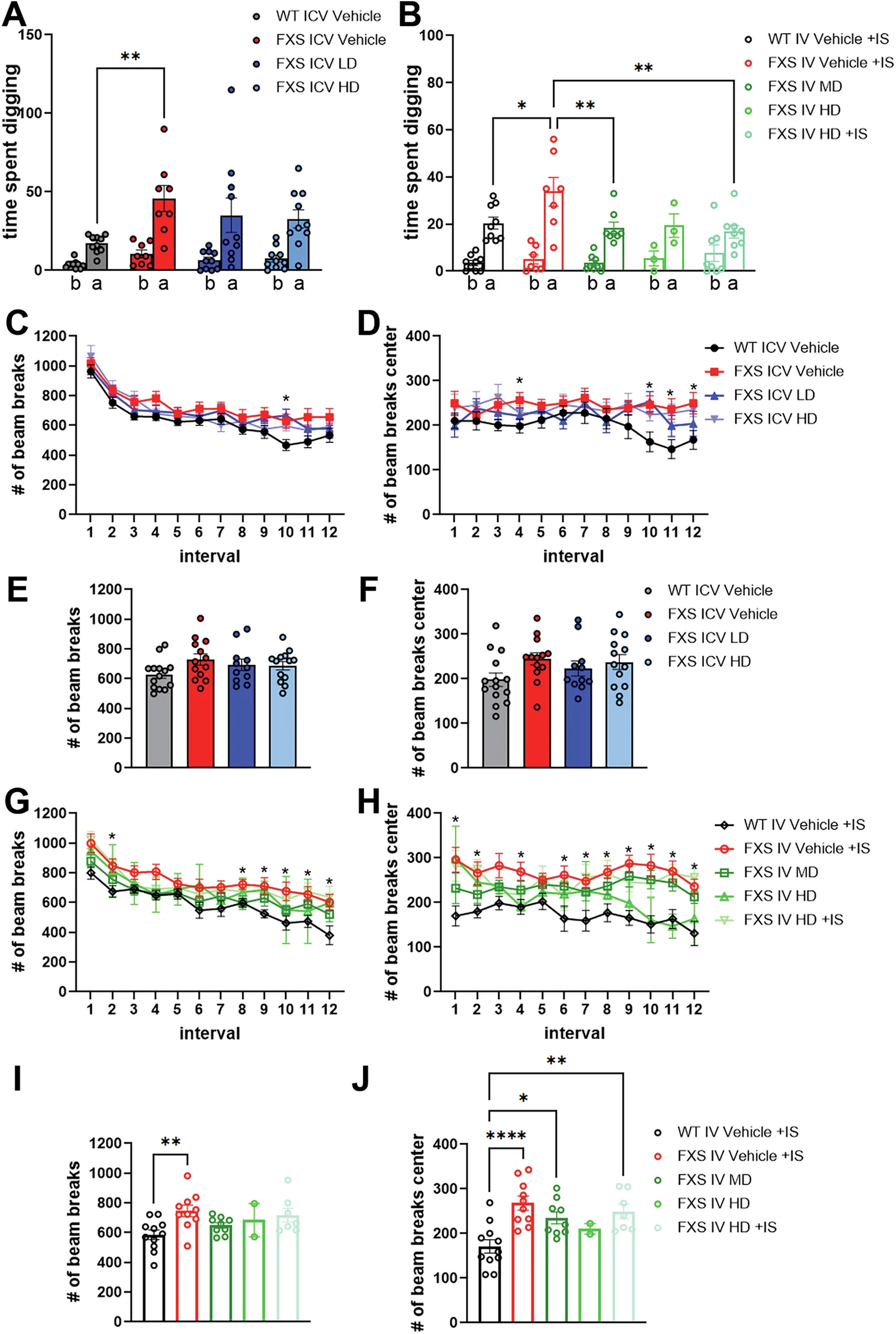
ICV or IV dosing of AAV/CAG-hFMR1 significantly reduces digging behavior and overall reduces open field activity. **A**, **B** Administration of AAV/CAG-hFMR1 reduces increased digging behavior in *Fmr1* KO mice after nest removal with more pronounced effects after IV dosing (**A** ICV dosing groups, repeated measures two-way ANOVA with Tukey’s post hoc tests, p(nest y/n)<0.0001, p(group)=0.033, p(interaction)=0.23, **p = 0.003, n(wt veh)=10, n(KO veh)=8, n(KO LD) = 10, n(KO HD) = 10; **B** IV dosing groups, repeated measures two-way ANOVA with Tukey’s post hoc tests, p(nest y/n)<0.0001, p(group)=0.099, p(interaction)=0.021, *p = 0.017, **p(veh-MD) = 0.0051, **p(veh-HD IS) = 0.0015, n(wt veh IS) = 9, n(KO veh IS) = 7, n(KO MD) = 8, n(KO HD = 3), n(KO HD IS) = 8). *b*, before nest removal, *a*, after nest removal (**C**–**F**) Open field activity in ICV dosing groups. (**C**, **E**) Overall time ambulatory is reduced over time during a 1-h monitoring period in all groups but not significantly different regardless of genotype or dosing group (**C** repeated measures two-way ANOVA with Dunnett’s post hoc tests, p(interval)<0.0001, p(group)=0.18, p(interaction)=0.10, *p(WT veh – KO veh)=0.043; **E** one-way ANOVA, p = 0.18; n(WT veh)=14, n(KO veh)=13, n(KO LD) = 11, n(KO HD) = 13). Time in center is increased in the last 15 min of the 1-h recording period in *Fmr1* KO mice compared with WT mice but not significantly altered after viral vector dosing (**D** repeated measures two-way ANOVA with Dunnett’s post hoc tests, p(interval)=0.066, p(group) =0.15, p(interaction)=0.18, *p(WT veh – KO veh)<0.05; **F** one-way ANOVA, p = 0.15; n as in **A**, **C**). **G**–**J** Open field activity in IV dosing groups. Overall time ambulatory is reduced over time during a 1-h monitoring period in all groups and significantly increased in vehicle-treated *Fmr1* KO mice compared with WT mice. No effect of viral vector administration was detected (**G** repeated measures two-way ANOVA with Dunnett’s post hoc tests, p(interval)<0.0001, p(group) =0.019, p(interaction)=0.69, *p(WT veh – KO veh)<0.05; **I** one-way ANOVA with Dunnet’s post hoc tests, *p* = 0.019; **p = 0.006, *n*(WT veh IS) = 11, *n*(KO veh IS) = 10, *n*(KO MD) = 9, n(KO HD) = 2, *n*(KO HD IS) = 7). Time in center is increased in vehicle IV dosed *Fmr1* KO mice compared with WT mice but not significantly altered after viral vector dosing (**H** repeated measures two-way ANOVA with Dunnett’s post hoc tests, p(interval)=0.074, p(group)=0.0004, p(interaction) =0.35, *p(WT veh – KO veh) <0.05; **J** one-way ANOVA, *p* = 0.0004; ****p < 0.0001, **p = 0.004, *p = 0.014; *n* as in **G**, **I**). Bar graphs and error bars represent mean with standard errors. Detailed statistics are shown in Supplementary Table 3. Successful B cell depletion is shown in Supplementary Fig. 3. *IS* immunosuppression.

Patients with FXS have difficulties adjusting to changes in their environment. To test how mice react to a change in their environment, we performed the nest removal/digging assay. Here, the time spent digging after removal of the nest from the home cage (“change in environment”) is used as a measure of their level of stereotyped motor behavior [36]. *Fmr1* KO mice spent more time digging after nest removal than their WT littermates, and overall, the *FMR1* gene therapy reduced digging behavior regardless of administration route or dose (Fig. 6A, RM 2-way ANOVA with Tukey’s post hoc tests, *p*(nest y/n)<0.0001, *p*(viral vector)=0.033, *p*(interaction)=0.23, **p = 0.003; Fig. 6B, *p*(nest y/n)<0.0001, *p*(viral vector)=0.099, *p*(interaction)=0.02, *p < 0.05, **p < 0.01). IV dosing was more effective for this phenotype and significantly reduced digging behavior to WT control levels. These results suggest that the *FMR1* gene replacement improves FXS-relevant behavioral problems. Importantly, *Fmr1* KO mice retained the phenotype of increased digging after immunosuppressant treatment (Fig. 6B). Previous studies have shown that *Fmr1* KO mice have increased mTOR signaling, and that daily dosing with 10 mg/kg rapamycin rescued select behaviors [37, 38]. The rapamycin dose applied here was lower (4 mg/kg, 3 times a week) and ended a few days before the digging assay, which could explain why the phenotype was not rescued.

Open field activity and time spent in center were increased in vehicle-treated *Fmr1* KO mice compared with WT littermates. No significant rescue with either of the treatment doses or routes was observed for total activity (Fig. 6C, E, G, I) or time in center (Fig. 6D, F, H, J), despite reduced total activity and time in center after viral vector dosing. Sample sizes and statistics for Figs. 5 and 6 are shown in Supplementary Table 3.

### AAV/CAG-hFMR1 reduces increased gamma EEG power in *Fmr1* KO mice

Given the variability in behavioral assays in *Fmr1* KO mice we observed, we next tested if the *FMR1* gene therapy rescues an objective, quantitative and translational electrophysiological phenotype, namely increased gamma frequency EEG power. We and others have previously shown that gamma EEG power is increased in both mouse models and patients [39–42]. To increase translational interpretability of the study, EEG power was measured in mice using in vivo multi-electrode arrays (MEA), an EEG technique that mimics scalp EEG recording in humans [41, 43].

**Fig. 7:**
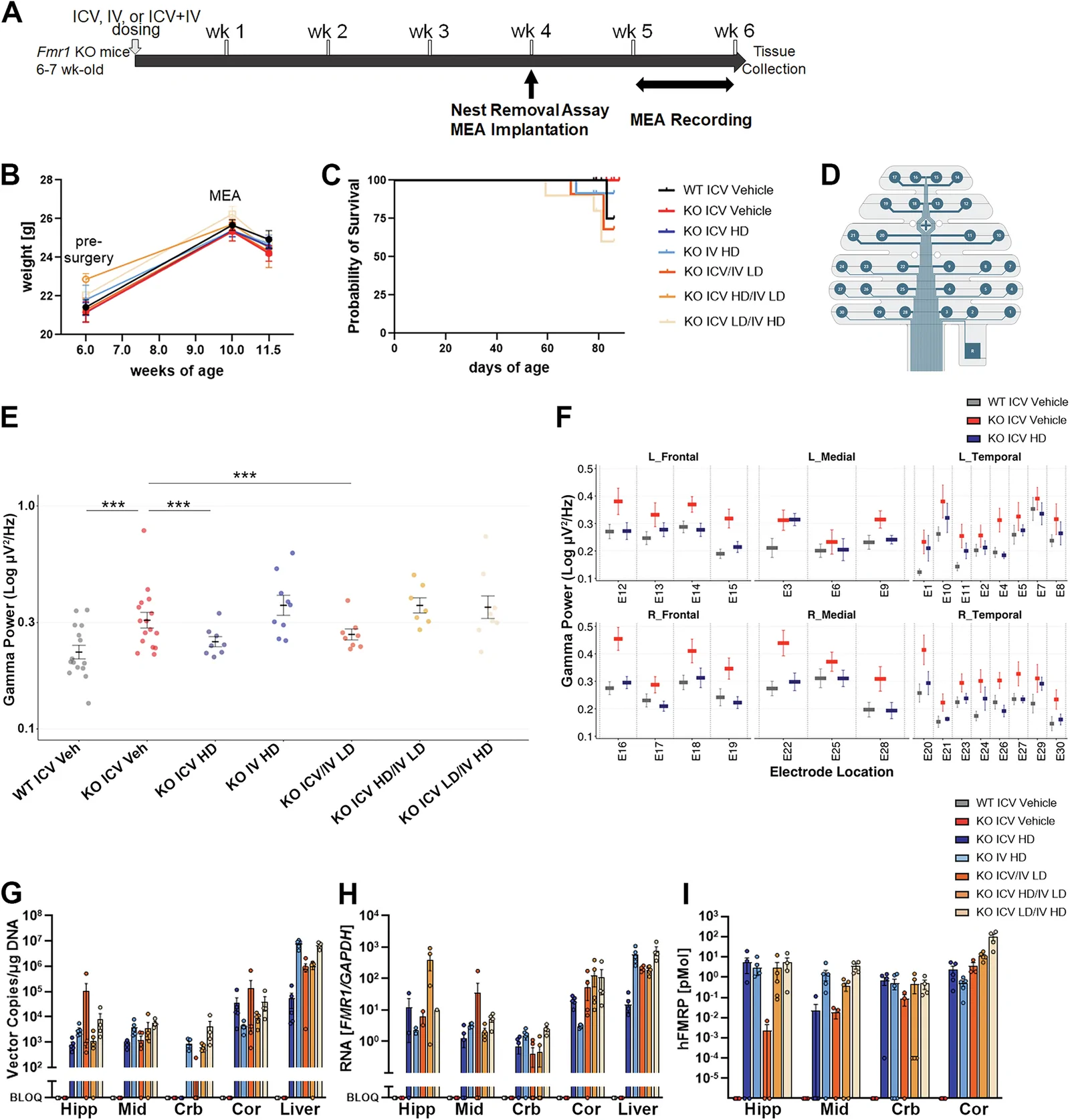
ICV dosing of AAV/CAG-FMR1 reduces increased gamma power in *Fmr1* KO mice. **A** Timeline of experiments. Dosing groups are shown in Table 2. **B** Mice in all groups gained weight as expected with a slight loss in weight after MEA surgery (mixed effects model, p(week)<0.0002, p(group)=0.6, p(interaction)=0.39, n(WT veh)=13, n(KO veh)=11, n(KO ICV HD) = 11, n(KO IV HD) = 11, n(KO ICV/IV LD) = 11, n(KO ICV HD/IV LD) = 10, n(KO ICV LD/IV HD) = 10). **C** No increased mortality was observed after dosing, and most deaths were directly associated with anesthesia or surgery (Log rank (Mantel-Cox) test, p = 0.071, n as in B). **D** Schematic of the MEA grid with channel numbers (*taken from NeuroNexus Technologies brochure*). **E** Absolute Gamma Power (µV^2/Hz) is increased in *Fmr1* KO mice and significantly reduced in by treatment group for the MEA recording (one-way ANOVA with Dunnet’s post hoc tests, p < 0.001, ***p < 0.001, n(WT veh)=15, n(KO veh)=16, n(KO ICV HD) = 8, n(KO IV HD) = 9, n(KO ICV/IV LD) = 8, n(KO ICV HD/IV LD) = 7, n(KO ICV LD/IV HD) = 9). Lines represent mean values, error bars represent standard errors, and dots represent individual mice. **F** Mean Absolute Gamma Power across different brain regions. Variability across channels is evident. The graph shows the mean absolute gamma power (µV²/Hz) per channel (*chan*), divided by the group. Each point represents the mean value per group, while the bars represent the standard error of the mean. The color of each point and error bar corresponds to the treatment group. Individual plots were generated for each region, with the x-axis scaled freely for each plot to account for varying numbers of channels per region. Quantification of viral vector copies by qPCR (**G**) *hFMR1* mRNA by RT-qPCR (**H**) and FMRP protein by Luminex analysis (**I**) in different brain regions and liver shows transduction in all expected areas with dual dosing routes generally leading to higher viral vector levels despite some variability in transduction and gene expression within each group (**G**–**I** mixed-effects models, p(tissue) < 0.001, *p*(group) < 0.001, p(interaction)<0.001, sample sizes between 4 and 6 for **G** 1 and 4 for **H** and 3 and 6 for **I**). Sample sizes and detailed statistics, including post hoc tests for **G**–**I** are shown in Supplementary Tables 4 and 5.

Mice were injected either ICV, IV or in combination using two different doses at 6–7 weeks of age. *Fmr1* KO and C57BL/6 control mice were directly purchased from The Jackson Laboratory. Mice were not treated with immunosuppressants, and IV doses were reduced from the previous study to avoid potential vector-induced mortality. The mice were weighed weekly for four weeks and tested in nest removal/digging assays at four weeks following injection, followed by MEA implantation surgeries and MEA baseline recording five to six weeks following surgery. Six weeks following injection, mice were collected for analysis of vector biodistribution in different brain regions and the liver. Dosing groups are shown in Table 2, and the timeline of experiments is shown in Fig. 7A.

**Table 2:** Groups and sample sizes of mice enrolled and tested for the MEA study.

Mouse Model	Treatment Group	Route	Dose	Dose Volume	Mice Enrolled	Mice Analyzed^a^
C57Bl/6	Vehicle	ICV	N/A	2 µl/ventricle	18	15^a^
*Fmr1* KO	Vehicle	ICV	N/A	2 µl/ventricle	16	16^a^
*Fmr1* KO	High Dose	ICV	1E11 vg/g est. brain mass	2 µl/ventricle	11	8
*Fmr1* KO	High Dose	IV	5E13 vg/kg	2.5 ml/kg	12	9
*Fmr1* KO	Low/low Dose	ICV + IV	ICV: 2E10 vg/g est. brain mass IV: 1E13 vg/kg	2 µl/ventricle 2.5 ml/kg	11	8
*Fmr1* KO	High/Low Dose	ICV + IV	ICV: 1E11 est. vg/g brain mass IV: 1E13 vg/kg	2 µl/ventricle 2.5 ml/kg	10	9
*Fmr1* KO	Low/High Dose	ICV + IV	ICV: 2E10 est. vg/g brain mass IV: 5E13 vg/kg	2 µl/ventricle 2.5 ml/kg	10	7

Some mice were not analyzed due to missed doses, surgery-related early death, or technical issues with the MEA cap. Brain weight was estimated as 0.4 g for 6-week-old mice [22].

^a^5 mice each were added from another study with a similar timeline.

All mice regardless of group gained weight as expected during the four weeks leading to surgery. Some weight loss was observed after MEA surgery in all groups, but mice continued to appear healthy (Fig. 7B). A few deaths occurred during the study, most of which were directly related to the surgical procedure. Only three deaths were not directly related to anesthesia (one each after ICV HD, ICV/IV LD, and ICV HD/IV LD), but no significant differences in survival were detected between groups (Fig. 7C). Sample sizes and statistics are shown in Supplementary Table 4A.

No differences between groups in nest removal/digging assays were detected in this cohort (Supplementary Fig. 9, Supplementary Table 4A). Because of the lack of a baseline difference between vehicle-treated *Fmr1* KO and WT mice, interpretation of lack of differences in the treatment group is not possible. The CCHMC mouse vivarium changed nesting material before this study started, which may have contributed to the loss of phenotype between this and the previous study. We speculate that this initial change in environment blunted the response to the change induced by removal of the material.

We next analyzed gamma EEG power as a quantifiable and translational biomarker using scalp multi-electrode arrays (MEA) mimicking human EEG. A schematic of an MEA with 32 channels is shown in Fig. 7D. Baseline MEA analyses showed that, as reported in previous studies [41, 43], gamma power was significantly increased in *Fmr1* KO mice compared with WT mice when averaged across all channels (Fig. 7E, one-way ANOVA with Dunnett’s post hoc tests, p < 0.001, ***p < 0.001). Gamma power was significantly reduced after high dose ICV and low dose IV/low dose ICV administration of the *hFMR1* viral vector. None of the other groups showed significant differences compared with vehicle-injected mice. When recordings from vehicle-treated *FMR1* KO, vehicle-treated WT mice, and high dose ICV AAV/CAG-hFMR1 treated mice were compared across different brain regions, differences in efficacy to reduce gamma power were observed (Fig. 7F), most likely due to differential spread of the viral vector across the brain. Sample sizes and statistics are shown in Supplementary Table 4B.

To assess viral vector biodistribution and transgene expression, we performed qPCR to quantify viral vector copies, qRT-PCR to quantify *hFMR1* mRNA, and a highly sensitive Luminex assay to quantify human FMRP [7] in different brain regions (cortex, hippocampus, midbrain, cerebellum) and liver. Viral vector copies were detected in all brain regions and organs tested. Combination doses tended to show higher numbers of viral vector copies. ICV injections yielded higher vector copy numbers in the cortex, whereas IV injections showed higher copy numbers in midbrain and liver (Fig. 7G, mixed-effects model with Tukey’s post hoc tests, p(tissue) <0.0001, p(viral vector) < 0.0001, p(interaction) < 0.0001, pairwise comparisons in Supplementary Table 5). Human *FMR1* mRNA expression had a similar distribution as the viral vector, with higher expression in the cortex and hippocampus after ICV injection compared with midbrain and liver, and overall higher expression levels after combination dosing (Fig. 7H, mixed-effects model, p(tissue)<0.0001, p(viral vector) = 0.0011, p(interaction) < 0.0001, pairwise comparisons in Supplementary Table 5). FMRP expression measured by a Luminex assay optimized to detect human FMRP [7] showed a similar distribution with high levels in cortex and hippocampus after ICV dosing, and higher levels in midbrain and cerebellum after IV dosing (Fig. 7I, mixed-effects model, p(tissue)=0.0025, p(viral vector)=0.0003, p(interaction)=0.0002, pairwise comparisons in Supplementary Table 5). Increased expression was observed in the cortex for combination dosing. Tissue from one mouse was not sufficient to do all three assays. Therefore, tissue for *FMR1* mRNA and viral vector copy quantification was taken from half of the mice whereas tissue for FMRP Luminex assays was taken from the other half of the cohort. This may explain the lack of clear correlation of *FMR1* mRNA and FMRP for some brain areas (e.g., hippocampus and midbrain) and illustrates variability in transgene biodistribution following ICV injection. Of note, FMRP expression in two brain regions critical for generating gamma waveforms, the cortex and the midbrain, is similar in the two groups that showed rescue of increased gamma EEG power (ICV HD and ICV/IV LD), supporting a dose-dependent rescue effect of hFMRP.

## Discussion

**Fig. 8:**
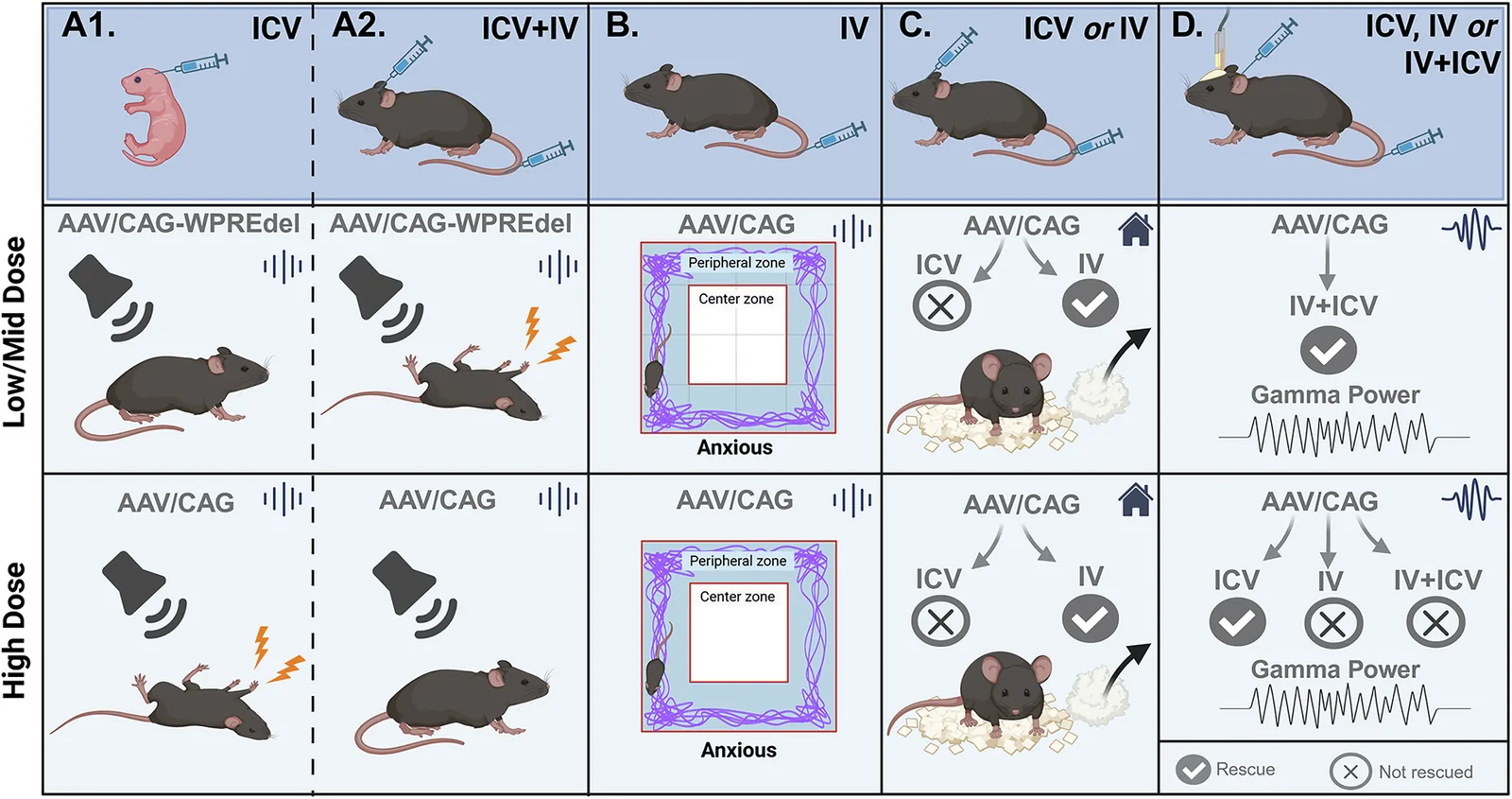
Overview of FMR1 gene therapy-mediated rescue based on dose, route, and assay. **A** Audiogenic seizures are rescued after dosing neonates ICV with a lower (AAV/CAG-WPREdel) but not a higher (AAV/CAG) expressing hFMR1 gene therapy vector (**A1**). By contrast, in adult mice, combined ICV and IV administration of a higher dose but not a lower expressing hFMR1 gene therapy vector rescues audiogenic seizures (**A2**). **B** IV administration of low or high dose hFMR1 gene therapy does not rescue altered open field behavior in adult mice. *ICV dosing could not be evaluated in this assay due to lack of a baseline phenotype in vehicle injected mice*. **C** Mid and high dose IV, but not ICV, administration of hFMR1 gene therapy normalizes increased digging after nest removal. **D** Low doses of combined ICV and IV injections as well as high dose ICV injection of hFMR1 gene therapy rescues increased gamma power, whereas neither high dose IV alone nor combinations of high and low doses of ICV and IV combined normalized this phenotype. In summary, this study suggests that there are different optimal routes and doses for FMR1 gene therapy dependent on the phenotype. Figure was prepared using *BioRender* software.

This study shows that an *FMR1* gene therapy using a viral vector suitable for human use improves several key deficits with high translational potential in male *Fmr1* KO mice. Depending on the route and dose, the gene therapy rescues specific deficits in three phenotypic domains present in mouse models and human patients: sensory hypersensitivity (audiogenic seizures), altered adaption to change (nest removal/digging), and altered brain activity (gamma EEG power). This gene therapy is successful when dosed at several ages, including neonatal (equivalent to *in utero* in humans), at weaning age (equivalent to toddler/kindergarten age in humans) and at 5–6 weeks of age (teenager/young adult in humans). Rescue effects are seen using two different dosing routes, ICV and IV, depending on the phenotype (Fig. 8). Biodistribution analyses support dose-dependent effects and suggest that a future FMRP gene therapy will require careful titration. In summary, this research supports the therapeutic promise of a gene replacement strategy in FXS.

An important aspect for developing successful therapeutic strategies in neurodevelopmental disorders like FXS is timing, i.e., the question of when treatment must start to be effective. Our studies show that re-expression of FMRP in mice at ages equivalent to 4-6 and 15-30 years in humans has the potential to rescue sensory hypersensitivity, stereotypic behavior, and excessive EEG gamma power. This suggests that certain FXS-related deficits are reversible or can be improved by re-expression of FMRP after large parts of brain development have already occurred. It also further supports the notion that apart from a role in neurodevelopment, FMRP can have acute functions in neurons and at synapses.

Another important consideration in the development of gene therapy is identifying the ideal dose to reach treatment efficacy. Our results assessing audiogenic seizure and EEG phenotypes suggest careful titration of the viral vector might be necessary. While AAV/CAG- hFMR1-WPREdel was more effective in reducing audiogenic seizures than AAV/CAG-hFMR1 after neonate dosing (Fig. 3B–D), the opposite was true for dosing in adolescent mice: AAV/CAG-hFMR1, but not AAV/CAG-hFMR1-WPREdel, was effective in reducing audiogenic seizures in adolescent mice (Fig. 4B–D). Expression analyses of *hFMR1* mRNA showed that the successful dose in neonates (AAV/CAG-hFMR1-WPREdel, Fig. 3E) led to comparable levels in the cortex as observed with the successful dose in adolescent mice (AAV/CAG, Fig. 4F) (~1.8-fold difference), whereas AAV/CAG dosing in neonates led to approximately 7-fold higher doses than dosing with AAV/CAG-hFMR1-WPREdel, suggesting FMRP overexpression led to reduced efficacy in rescuing audiogenic seizures. The importance of dosing levels was supported by the EEG analyses (Fig. 7). Here, high dose ICV and a combination of low dose ICV and IV both significantly reduced gamma EEG power, whereas neither high dose IV nor combinations of low and high doses of ICV and IV were effective (Fig. 7E). We speculate that under- or overexpression of FMRP in the cortex may have contributed to the lack of EEG rescue in some dosing groups. Notably, the two dosing paradigms that did rescue the MEA phenotype led to comparable FMRP expression levels in the cortex (~1.5-fold difference), whereas all other dosing paradigms led to substantially lower (IV HD, ~0.2-fold) or higher (IV/ICV combination, between ~3.5- and 30-fold) levels in the cortex. Combined, these results suggest that either too little or too much FMRP expression can lead to suboptimal therapeutic efficacy. A previous study showed that overexpression of FMRP by ~200% leads to hyperactivity, suggesting that too much FMRP can negatively affect brain function [15]. It is important to note that, except for high-dose IV in 3-week-old mice (Fig. 5E), none of our dosing paradigms led to adverse effects in mice despite the wide range of expression levels described above. The adverse effects we observed in the high dose IV group were reversed with immune suppression, suggesting an immune response that could have been elicited by either the viral vector or the transgene. Western blot analyses showed that even with a mid-dose IV injection of AAV/CAG-hFMR1 FMRP expression was on average 3.8fold higher than endogenous FMRP in untreated wild type mice (Supplementary Fig. 4); however, mice gained weight as expected (Fig. 5C) and histopathological analyses revealed no or only sporadic, mild hepatocytic degeneration, further supporting that an immune response rather than liver toxicity was cause of death. Future studies should carefully evaluate and monitor liver function, given that lack of FMRP in mice leads to higher susceptibility to hepatitis-induced long-term liver damage [44]. It is conceivable that overdosing of FMRP in the brain of patients is unlikely given the size of the human brain and the consequential challenges of sufficient expression levels of transgene. In summary, these results warrant further investigation into the most effective and safe dosing paradigms. As AAV9 has tropism for liver and heart, future AVV9-based gene vectors for FXS could include sequences for organ-specific microRNA-mediated silencing in heart or liver [45, 46].

A longstanding problem in the field of FXS is that the many rescue strategies that improved phenotypes in the *Fmr1* KO mouse model did not lead to clear improvements in clinical studies [47]. The reasons for this lack of translation are most likely manifold, including challenges in clinical trial design and limitations of the mouse model and the assays used [48]. While mouse behavior is an important approach to measure functional consequences of interventions, the translational usefulness may be limited. It is unclear how a subtle improvement in learning or memorizing spatial navigation in mice translates into improvement in complex behaviors in patients. Moreover, the *Fmr1* KO behavioral phenotype is highly variable as described before [33, 34] and shown in this study. To improve the translational value of our studies for clinical development we therefore tested a quantifiable robust electrophysiological phenotype that is observed in mice and humans, increased gamma EEG power [41–43, 49, 50]. Importantly, we used 32 channel MEA recording that mimics human scalp EEG, further increasing the translational value of this approach. Using this approach, we show that the *hFMR1* gene therapy vector rescues increased gamma power in *Fmr1* KO mice. Several studies support the relevance of gamma power oscillations for brain function in healthy individuals [51]. Small scale studies in humans with FXS support correlative associations between increased gamma power and cognitive and neuropsychiatric impairments [52, 53]. Moreover, elevated or altered gamma power is linked to sensory hypersensitivity, which is a hallmark of Fragile X syndrome [54]. Combined, these studies support the physiological relevance of altered gamma oscillations for the FXS phenotype. Future studies should investigate the causal relationships between rescue of EEG phenotypes and behavioral improvement in FXS to fully assess the potential of increased gamma power as relevant outcome measure for FXS.

Mechanistically, gamma oscillations have been proposed as a biomarker for excitatory/inhibitory (E/I) balance [55]. Rescue of increased gamma EEG power could thus suggest that the *hFMR1* gene therapy normalized altered E/I balance in *Fmr1* KO mice. Gamma oscillations are mediated by parvalbumin-positive fast-spiking interneurons [56], which have been shown to be dysregulated in FXS [57], further supporting the interpretation that normalization of gamma oscillations may be due to restoration of normal E/I balance. Future studies should use electrophysiological approaches to directly assess interneuron function after *hFMR1* gene therapy.

Overall, our study suggests that ICV or a combination of ICV/IV injections are most effective. Importantly, ICV injections led to the highest accumulation of FMRP in the cortex, whereas IV dosing distributed AAV9 vector-encoded FMRP more broadly in all brain regions analyzed (Fig. 2E–J). This suggests that for a comprehensive improvement of FXS-associated deficits, a combination therapy of ICV and IV administration may be needed. Several intra-CSF delivery methods of viral vectors in humans were evaluated, including intrathecal, intracisternal magna and ICV administration [15]. Therefore, albeit more invasive than IV alone, combination of ICV and IV dosing is feasible in humans. An ICV/IV combination route of a viral vector is currently being tested for a patient with a neurodegenerative disorder caused by *ASPA* mutations (NCT05317780).

A limitation of our study is that we did not detect baseline differences between vehicle-treated *Fmr1* KO and WT mice in most behavioral assays, making it difficult to precisely assess the behavioral effect of gene therapy. Given that mice were dosed four weeks before most behavioral assessments started, it is unlikely that this lack of phenotypes is caused by the ICV surgery or retro-orbital IV injection. We speculate that the main reason is the variability and inconsistency of behavioral phenotypes in *Fmr1* KO mice, which have been described but may also be underreported [34]. Other limitations include that not all studies used littermate WT controls, which may have contributed to the loss of the baseline phenotype in nest removal behavior. We also did not test for rescue of molecular phenotypes commonly observed in FXS, such as dysregulation of metabotropic glutamate receptor (mGluR)-induced protein synthesis or altered intracellular signaling [2]. In pilot studies, we quantified phosphorylation of Akt in hippocampal lysates, but the results were inconsistent. We assume that the stress induced by the behavioral testing, for example the Morris Water Maze, which was run last, could have caused these inconsistencies [58]. Future studies examining mGluR-dependent synaptic protein synthesis and intracellular signaling will be important to confirm normalization of hallmark molecular phenotypes in *Fmr1* KO mice. Moreover, it will be interesting to identify the neuronal subpopulations and brain circuits involved in specific phenotypes of *Fmr1* KO mice by analyzing synaptic markers to identify neuronal subtypes re-expressing FMRP and by brain-regionally restricted re-expression of human FMRP.

We observed increased mortality and moribundity in mice that were administered a high dose of viral vector IV (1 × 10^14^ vg/kg, ~1.5 × 10^12^ per mouse). Using the same dose and route in combination with an immunosuppression treatment did not lead to increased mortality. It is thus likely that the high dose administered IV caused an immune reaction in the mice. Further studies, including a comprehensive hematological assessment, are needed to fully understand the increased mortality in this group. Nevertheless, the observation that the adverse effects could be prevented with immune suppression supports the conclusion that the FMR1 gene therapy can be safely administered in humans. It is important to note that this dose (~1.5 × 10^12^ per mouse) was similar to other reported AAV9 studies with IV dosing in mice where no adverse effects were shown [59]. Therefore, the potential immune reaction observed with high dose IV administration of *FMR1* gene therapy could be related to the expressed transgene FMRP and not caused by the viral capsid. Recent studies using peripheral blood of patients with FXS suggest that FMRP is expressed in low levels even in patients with fully mutated (CGG repeat number over 200) and hypermethylated *FMR1* gene, and therefore *Fmr1* KO mice, in which the gene is deleted, do not perfectly replicate the molecular phenotype [7, 60]. It is intriguing to speculate that a patient subgroup with low levels of FMRP expression, in which the transgene is not expected to cause an immune reaction, is specifically amenable to the *FMR1* gene therapy.

In conclusion, here, we show that an *FMR1* viral gene therapy designed following gene therapies approved for human use rescues FXS-relevant highly translational phenotypes in *Fmr1* KO mice and supports that gene supplementation is a promising therapeutic strategy in FXS.

## Supplementary information

Supplementary Information

## Data Availability

The datasets used and/or analyzed in this study are available from the corresponding authors upon request.
