# Supplementary material for: FMR1 gene therapy restores translationally relevant phenotypes in a mouse model for fragile X syndrome
Source: Gene Ther. 2026 Jul 10;33(5):515–31. doi: 10.1038/s41434-026-00630-4 (PMC13585560; doi:10.1038/s41434-026-00630-4)
Supplement: Supplementary file 1 [file 41434_2026_630_MOESM1_ESM.pdf]

Supplementary Information for Manuscript

FMR1 Gene Therapy Restores Translationally Relevant Phenotypes in a Mouse Model for Fragile X Syndrome

RK Lacher[1], K Henson[2], LN Wathen[1,3], C Jones[2], T Arnold[2], MR Rice[3], HM Carles[3], AR White[3], E Ramsuchit[2], D Murrey[2], R Raig[2], A Fisher[4], K Bucher[1], GC Westerkamp[1], AL Fritz[3], BM Gollaway[3], S Piloto[1], D Dismuke[2], JE Robinson[4,5], MT Williams[3,5], CV Vorhees[3,5], EK De Silva[2], D Tiwari[3,5]*, CA Erickson[1,6]*, EV Pedapati[1,3,6]*, C Gross[3,5]*

[1]Division of Child and Adolescent Psychiatry, Cincinnati Children's Hospital Medical Center, Cincinnati, OH, USA

[2]Forge Biologics, Grove City, OH, USA

[3]Division of Neurology, Cincinnati Children's Hospital Medical Center, Cincinnati, OH, USA

[4]Division of Experimental Hematology and Cancer Biology, Cincinnati Children's Hospital

[5]Department of Pediatrics, University of Cincinnati College of Medicine, Cincinnati, OH, USA

Medical Center, Cincinnati, OH, USA

[6]Department of Psychiatry, University of Cincinnati College of Medicine, Cincinnati, OH, USA

*correspondence should be addressed to

C Gross, christina.gross@cchmc.org

EV Pedapati, ernest.pedapati@cchmc.org

CA Erickson, craig.erickson@cchmc.org

D Tiwari, durgesh.tiwari@cchmc.org

Content

This supplementary information contains 9 supplementary figures, 5 supplementary tables as well as supplementary methods.

Supplementary Figure 1 – related to Figure 2, western blot analyses of liver and heart

Supplementary Figure 2 – related to Figure 3, comparison of audiogenic seizures and biodistribution in mice with or without visible backflow of viral vector during injections

Supplementary Figure 3 – related to Figures 5 and 6, examples and quantification of immunosuppression

Supplementary Figure 4 – related to Figures 2, 5 and 6, comparison of endogenous and transgene FMRP expression in the liver

Supplementary Figure 5 – related to Figures 5 and 6, nesting behavior

Supplementary Figure 6 – related to Figures 5 and 6, Morris Water Maze Acquisition

Supplementary Figure 7 – related to Figures 5 and 6, Morris Water Maze Reversal

Supplementary Figure 8 – related to Figures 5 and 6, social interaction

Supplementary Figure 9 – related to Figure 7, nesting removal assay of mice in the MEA study

Supplementary Table 1 – related to Figure 2, hFMRP expression levels in different organs after dosing

Supplementary Table 2 – sample sizes and statistics for Figures 3 and 4

Supplementary Table 3 – sample sizes and statistics for Figures 5 and 6

Supplementary Table 4 – sample sizes and statistics for Figure 7 B-F

Supplementary Table 5 – sample sizes and statistics for Figure 7G-I

Supplementary Methods – nesting behavior, social preference assay, Morris water maze test

Supplementary Figure 1

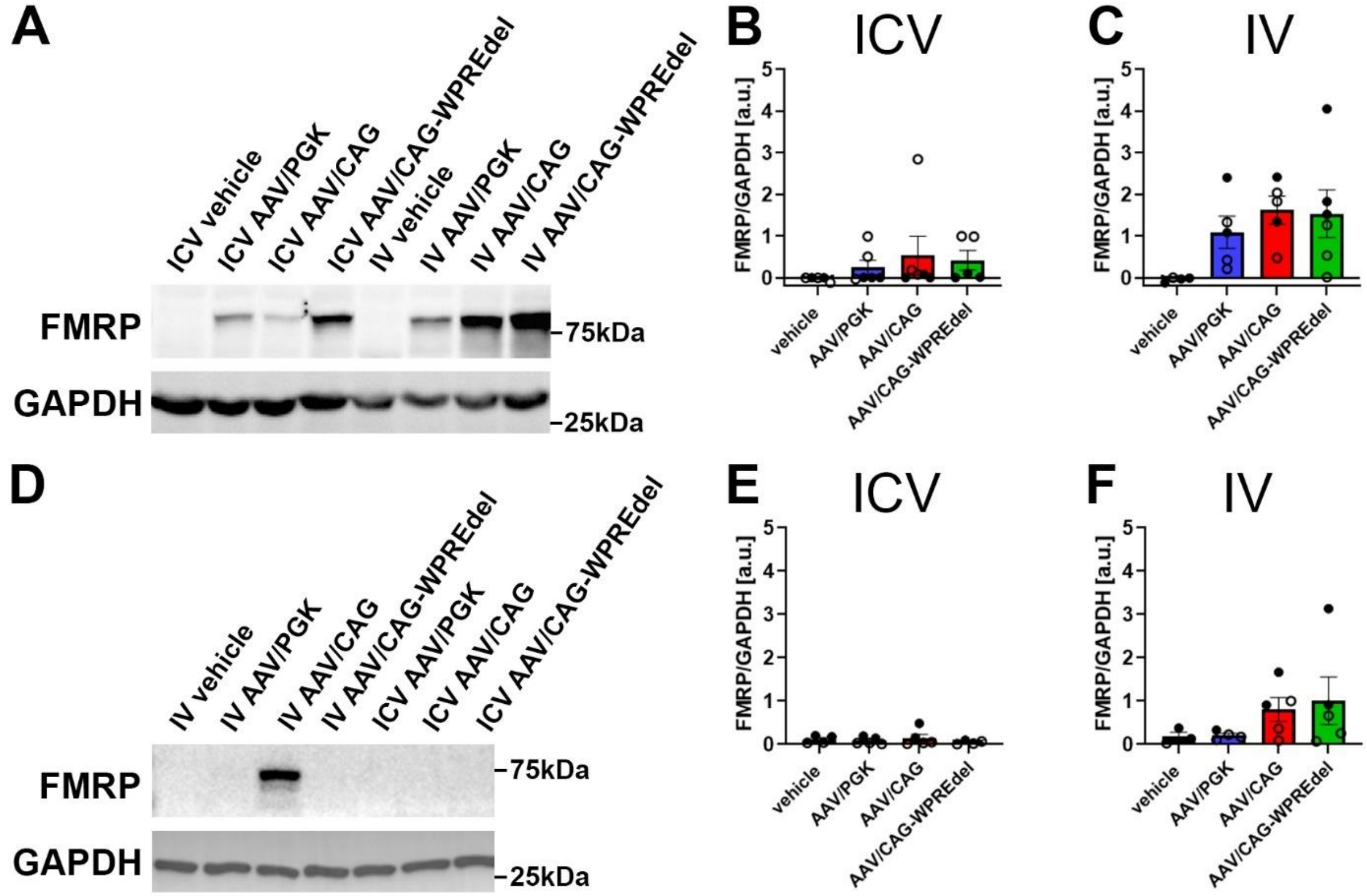

Supplementary Fig. 1 (Related to Figure 2): Western blot analysis of transgenic FMRP expression in liver and heart of mice dosed ICV or IV with three vector candidates or vehicle. (A-C) Western blot analysis of liver lysates shows that both IC and IV dosing of all three AAV/hFMR1 vector candidates leads to expression of hFMRP in the liver. As expected, IV dosing (C) leads to higher hFMRP expression levels than ICV dosing (B), and the AAV/PGK viral vector shows lowest expression levels. Example western blot shown in A. **(D-F)** Western blot analysis of heart lysates shows that IV dosing (F) leads to hFMRP expression in the heart, whereas ICV dosing shows no or marginal hFMRP expression in the heart (E). Again, the AAV/PGK vector showed the lowest expression levels. Example western blot shown in D. Bar graphs and error bars represent mean with standard errors.

Supplementary Figure 2

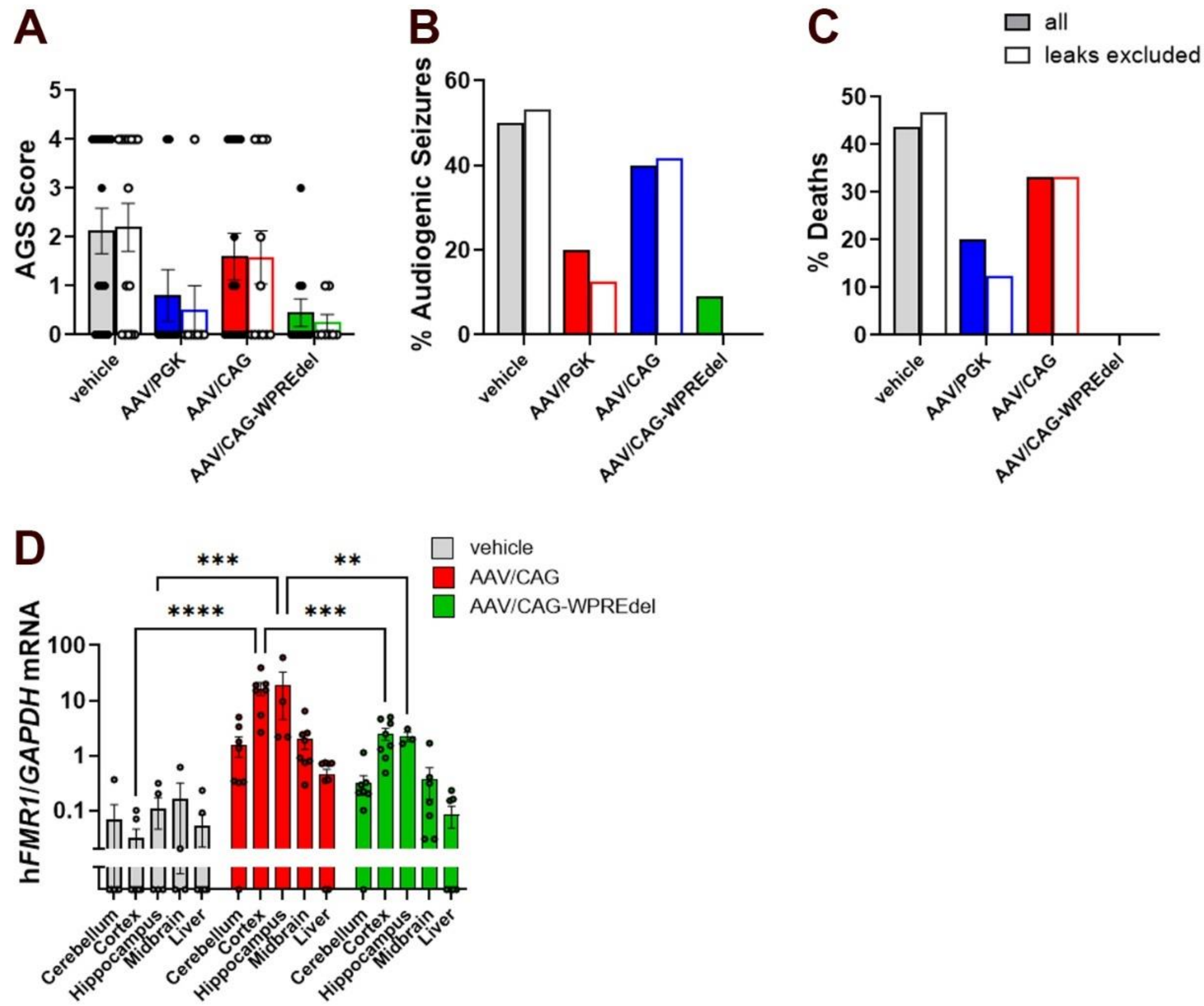

Supplementary Figure 2 (related to Figure 3): Minor leaks during surgery do not affect the efficacy of viral vectors to reduce audiogenic seizures. (A-C) No significant differences in audiogenic seizure score (A), occurrence (B) or audiogenic seizure-related deaths (C) were observed when mice with visible minor leaks during surgery were excluded (A, two-way ANOVA, p(leak)=0.75, p(viral vector)=0.0009, p(interaction)=0.98; B, two-way ANOVA, p(leak)=0.43, p(viral vector)=0.005; C, two-way ANOVA, p(leak)=0.64, p(viral)=0.0024). **(D)** Likewise, *hFMR1* mRNA biodistribution in different brain regions and liver was comparable when mice with leaks were excluded (see Fig. 3E) with overall higher expression levels after AAV/CAG dosing and strongest expression in cortex and hippocampus (two-way ANOVA, p(organ)=0.0013, p(viral)<0.0001, p(interaction)=0.0015). Between 0 and 3 mice per group were removed due to visible leaks depending on the assay. Bar graphs and error bars represent mean with standard errors. Sample sizes and detailed statistics are shown in Supplementary Table 2.

Supplementary Figure 3

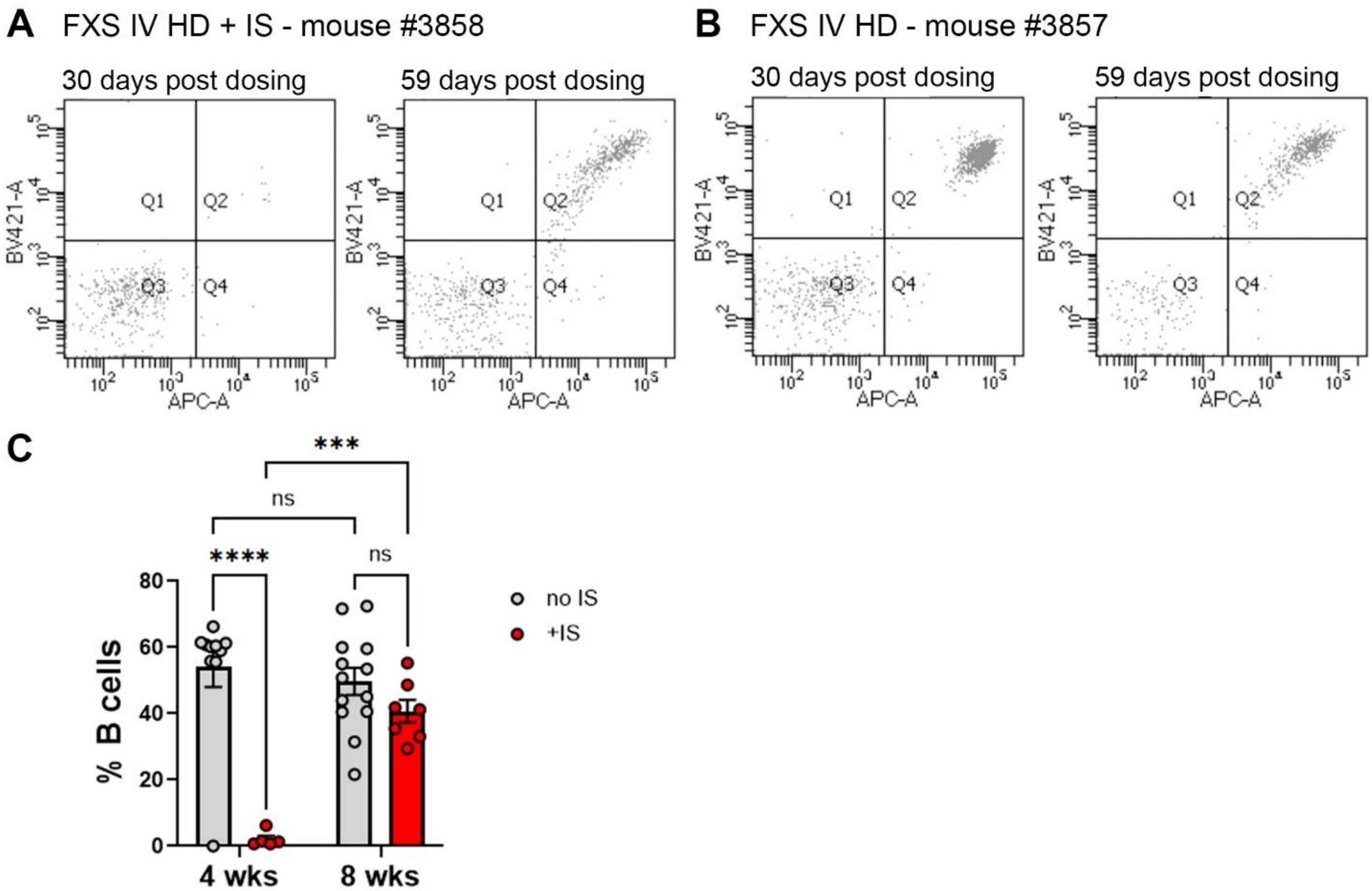

Supplementary Figure 3 (related to Figures 5 and 6): Anti-CD20 treatment leads to transient depletion of B cells. (A,B) Example FACS results of peripheral blood samples from a mouse that received anti-CD20 treatment at the time of viral vector dosing (A) and a mouse that was not treated with anti-CD20 antibodies (B). Blood samples were taken at ~4 weeks (30 days) and ~8 weeks (59 days) after dosing and analyzed by FACS using a gating strategy to select for singlets, live cells (7AAD (650nm) negative) > CD45 positive (490 nm) > B220 and CD19 positive (421 nm and 660 nm). **(C)** Quantification using FACSDiva™ software (BD Bioscience) showed significant depletion of B cells in immunosuppressed mice 4 weeks after treatment which was restored 8 weeks later (two-way ANOVA with Fisher's LSD, p(time)=0.0045, p(immunosuppression)<0.0001, p(interaction)=0.001, ****p<0.0001, ***p=0.0004). Immunosuppressed or non-immunosuppressed mice from several dosing groups were combined. Bar graphs and error bars represent mean with standard errors. Sample sizes and detailed statistics are shown in Supplementary Table 3.

Supplementary Figure 4

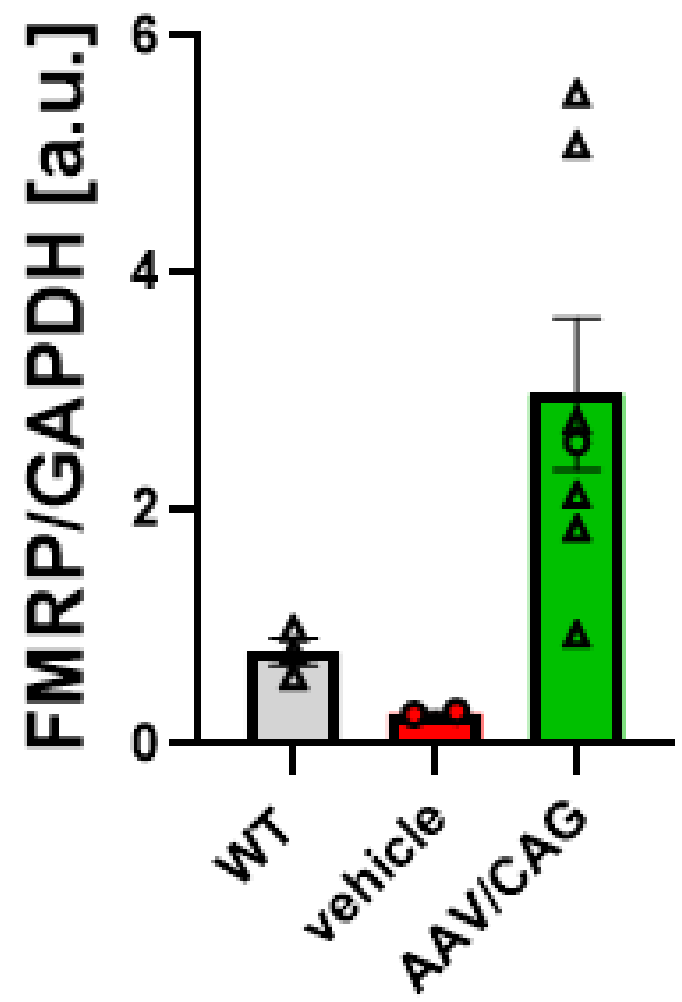

Supplementary Figure 4 (related to Figures 2, 5 and 6): IV dosing of AAV/CAG leads to 3.8fold higher expression of recombinant FMRP than endogenous FMRP in the liver. Liver lysates from wild type mice (grey, n = 3), and *Fmr1* KO mice IV dosed with vehicle (red, n = 2) or AAV/CAG-hFMR1 ($3x10^{13}$ vg/kg, green, n = 7) were analyzed for FMRP expression with an antibody detecting both human and mouse FMRP followed by an antibody detecting mouse GAPDH (one-way ANOVA with Tukey's multiple comparison tests, p=0.047, pairwise comparisons: p(AAV/CAG-hFMR1 versus vehicle) = 0.089, p(AAV/CAG-hFMR1 versus WT) = 0.11, p(vehicle vs WT) = 0.914). Western blots were done using Jess Simple Western Blotting (*biotechne*). Circles are male, triangles are female mice. One female wild type mouse was pregnant at the time of tissue collection; however, because FMRP levels were very consistent between the three tested wild type mice, we do not think this affected the results.

Supplementary Figure 5

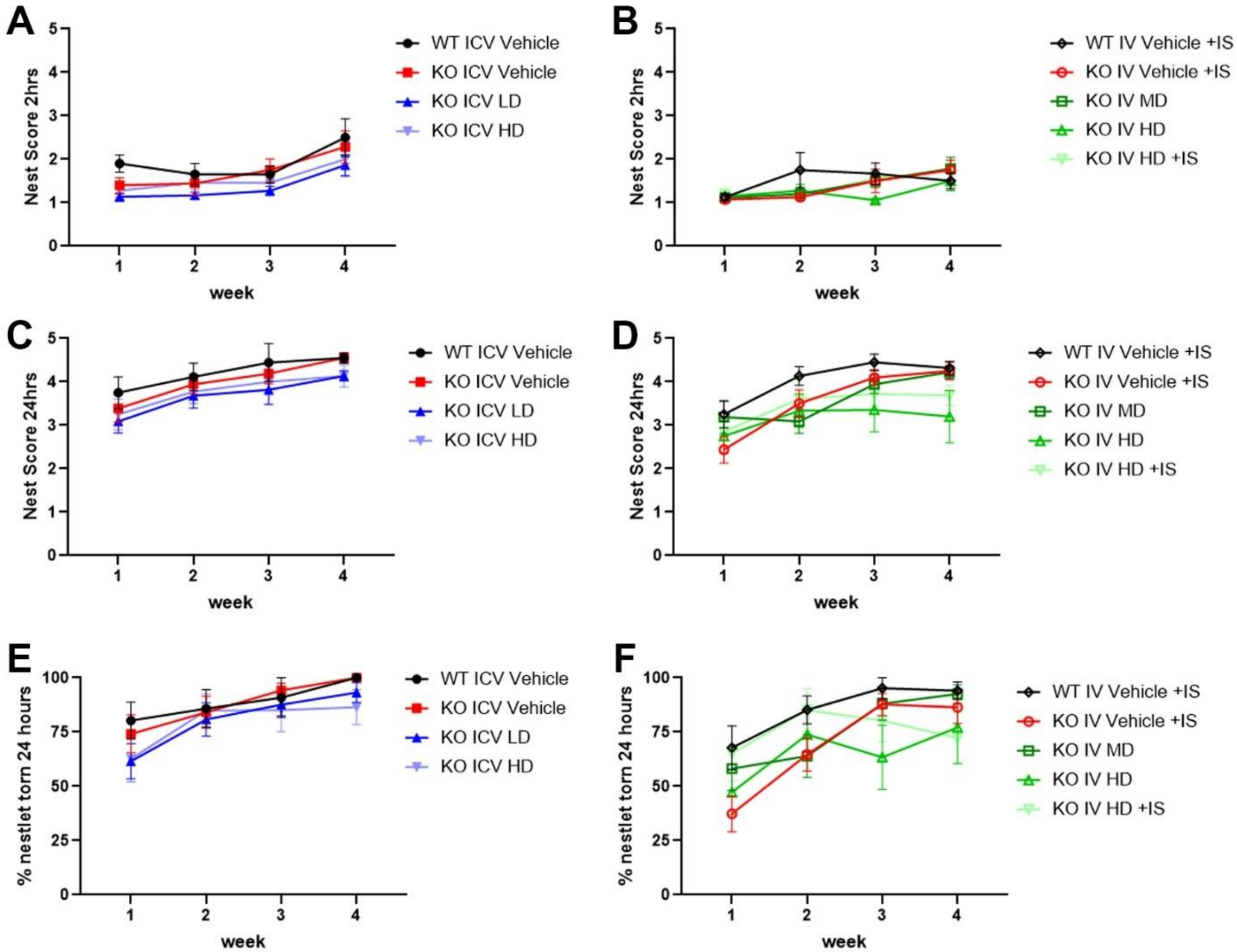

Supplementary Figure 5 (related to Figures 5 and 6): No baseline differences in vehicle-treated WT and *Fmr1* KO mice in nesting behavior. (A,B) Nesting scores of ICV (A)- and IV (B)-treated mice do not differ two hours after nestlet placement across the 4 weeks after dosing. **(C,D)** Nesting scores of ICV (C)- and IV (D)-treated mice do not differ twenty-four hours after nestlet placement across the 4 weeks after dosing. **(E,F)** Percent nestlet torn of ICV (A)- and IV (B)-treated mice do not differ two hours after nestlet placement across the 4 weeks after dosing. N between 8 and 10 for ICV-injected mice and 6-13 for IV-injected mice. Mixed effect analyses showed significant effects of week ($p<0.0001$) for all analyses indicating that all mice improved over time in nesting behavior. Group size varies for measurement and week, ranges are given: n(WT ICV Vehicle) = 11-13, n(KO ICV Vehicle) = 8, n(KO ICV LD) = 11, n(KO ICV HD) = 8-9,

n(WT IV Vehicle +IS) = 6-11, n(KO IV Vehicle +IS) = 7-9, n(KO IV MD) = 9-10, n(KO IV HD)=6-7, n(KO IV HD +IS) = 7-12.

Supplementary Figure 6

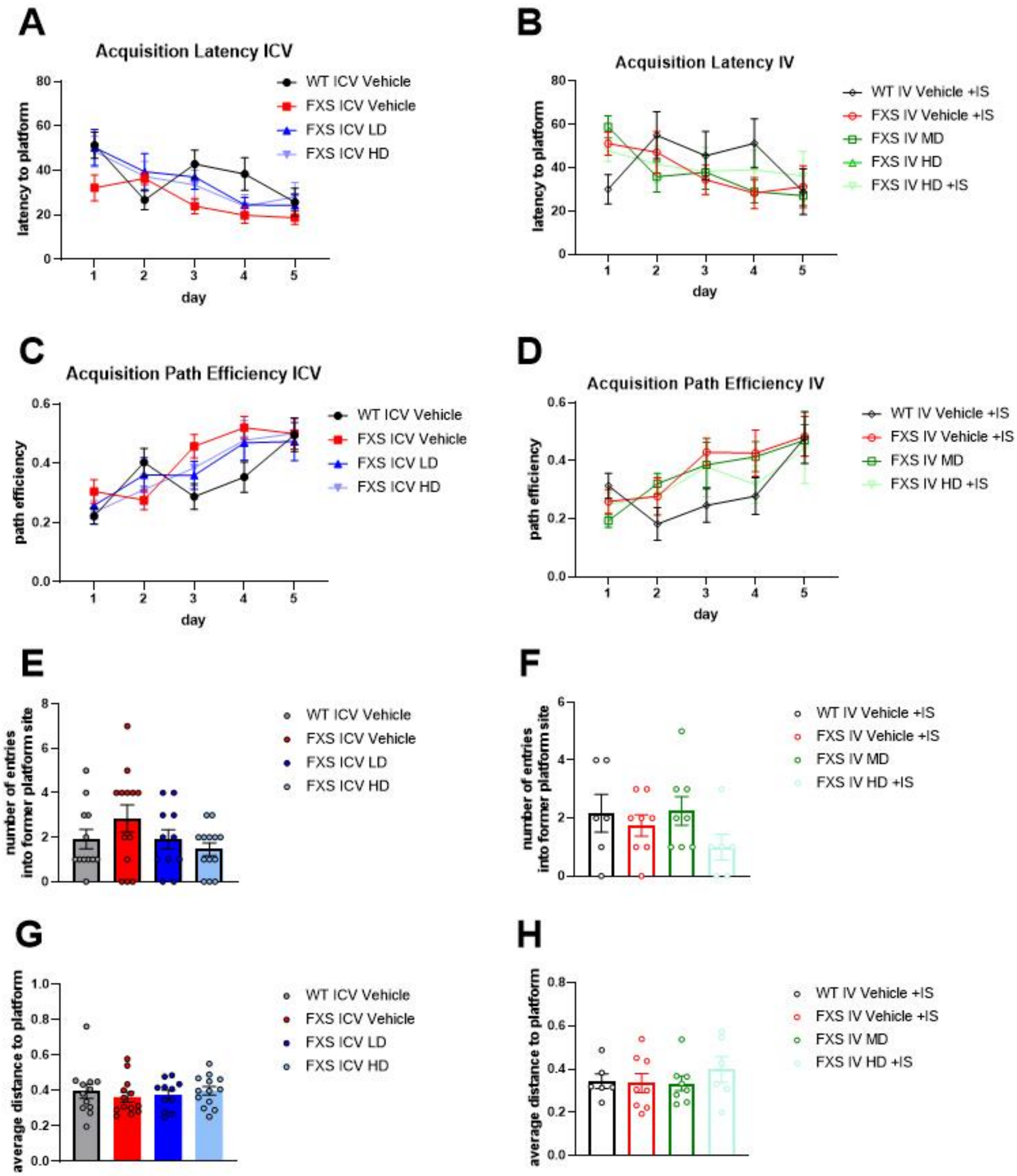

Supplementary Figure 6 (related to Figures 5 and 6): No clear differences in Morris water maze acquisition training or acquisition probe trial regardless of genotype or treatment.

Results are shown separately for ICV (A, C, E, G)- and IV (B, D, F, H)-treated mice. All mice

improved over time, and latency to platform (A,B) or path efficiency (C, D) were not significantly different between groups (mixed-effects model, p(interval)<0.0001, p(group)>0.05, p(intervalXgroup)=0.05 (A), 0.0033 (B), 0.07(C), and 0.349 (D)). Note that the WT groups showed unexpected acquisition pattern (no consistent improvement in both cohorts) leading to significant interactions in some instances; however, the biological relevance of these differences is unclear. During probe trial, number of entries into correct former platform site (E,F), or distance to former platform (G,H) are not significantly different between groups (1-way ANOVA, p>0.05). n(WT ICV Vehicle) = 13, n(KO ICV Vehicle) = 14, n(KO ICV LD) = 14, n(KO ICV HD) = 16, n(WT IV Vehicle +IS) = 6, n(KO IV Vehicle +IS) = 9, n(KO IV MD) = 8, n(KO IV HD +IS) = 7. Group KO IV HD was left out because of too low n due to early mortality.

Supplementary Figure 7

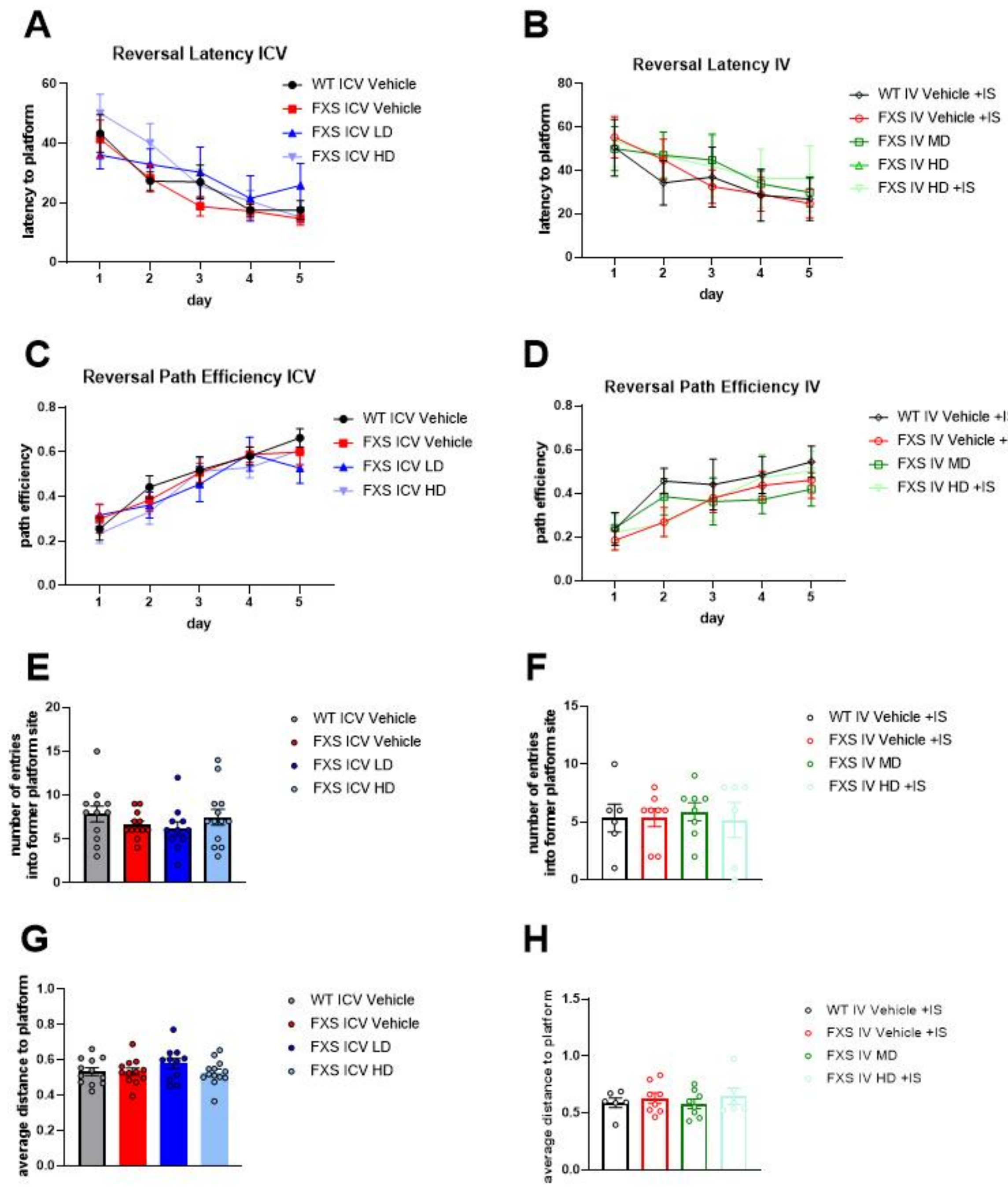

Supplementary Figure 7 (related to Figures 5 and 6): No clear differences in Morris water maze reversal training or reversal probe trial regardless of genotype or treatment. Results are shown separately for ICV (A, C, E, G)- and IV (B, D, F, H)-treated mice. All mice improved over time, and latency to platform (A,B) or path efficiency (C, D) were not significantly different

between groups (mixed-effects model, p(interval)<0.0001, p(group)>0.05, p(dayXgroup)=0.049, >0.05 for B-D). The significant interaction in A is caused by an unusual pattern in the WT group (performed worse on day 3 than day 2), and thus the biological relevance is unclear. During probe trial, number of entries into former platform site (E,F) or distance to former platform (G,H) were not significantly different between groups (1-way ANOVA, p>0.05). n(WT ICV Vehicle) = 12, n(KO ICV Vehicle) = 13, n(KO ICV LD) = 11, n(KO ICV HD) = 13, n(WT IV Vehicle +IS) = 6, n(KO IV Vehicle +IS) = 8, n(KO IV MD) = 8, n(KO IV HD +IS) = 6. Group KO IV HD was left out because of too low n due to early mortality.

Supplementary Figure 8

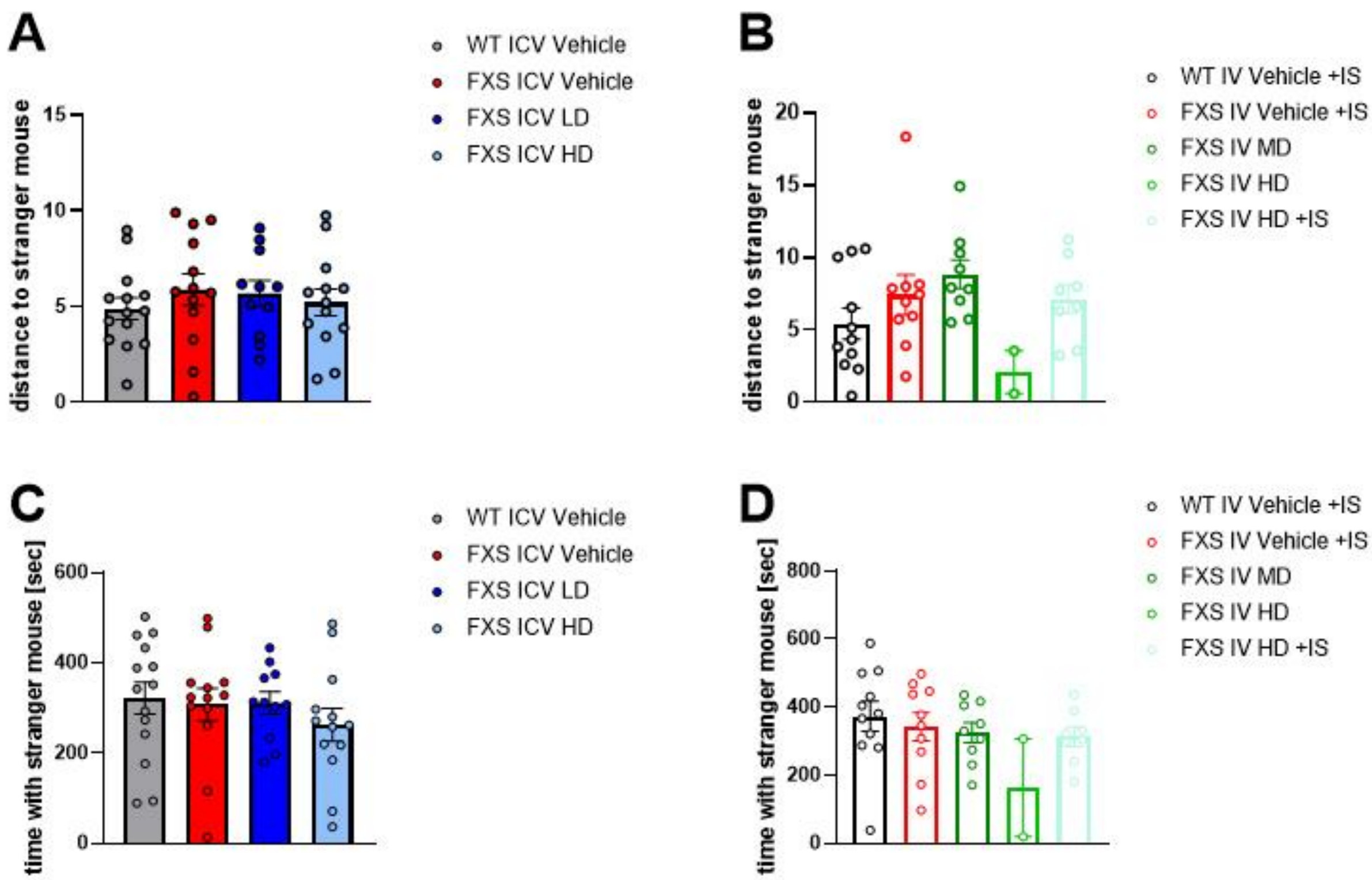

Supplementary Figure 8 (related to Figures 5 and 6): No differences in distance to stranger mouse or time spent with stranger mouse in the social preference assay regardless of genotype or treatment. (A,B) Distance to stranger mouse of ICV (A)- and IV (B)-treated mice. **(C,D)** Time spent with stranger mouse of ICV (C)- and IV (D)-treated mice. One-way ANOVAs all p>0.05. n(WT ICV Vehicle) = 14, n(KO ICV Vehicle) = 13n(KO ICV LD) = 11, n(KO ICV HD) = 13, n(WT IV Vehicle +IS) = 11, n(KO IV Vehicle +IS) = 10, n(KO IC MD) = 9, n(KO IV HD) = 2, n(KO IV HD +IS) = 8. Highest value in group KO IV Vehicle +IS in B was identified as a statistical outlier using the ROUT method but did not change overall results or conclusions.

Supplementary Figure 9

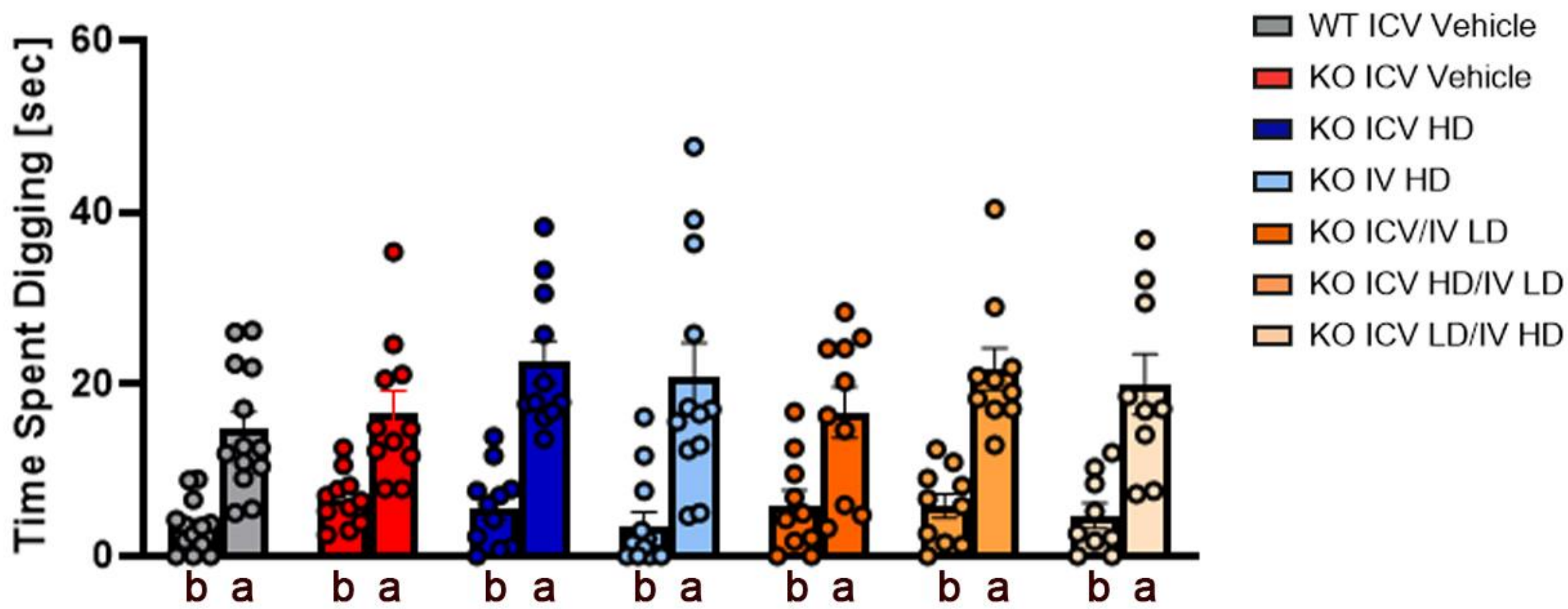

Supplementary Figure 9 (related to Figure 7): No baseline differences and no effects of AAV/CAG-hFMR1 on digging behavior after nest removal in mice dedicated for MEA analysis. Time spent digging before (*b*) or after (*a*) nest removal did not differ between vehicle-injected wild type or *Fmr1* KO mice, making interpretation of results after AAV/CAG-hFMR1 dosing difficult (two-way ANOVA, p(nest y/n)<0.0001, p(group)=0.529, p(interaction)=0.168). Bar graphs and error bars represent mean with standard errors. Sample sizes and detailed statistics are shown in Supplementary Table 4A. *b*, before nest removal, *a,* after nest removal

Supplementary Table 1: Western Blot Analysis of hFMRP Expression

Mouse	Sex	Injection	Heart	Liver	Kidney	Ovaries	DRG	Muscle	Lung
**3388**	Female	IV BLZ-21-031	-	-	-	-	-	-	-
**3390**	Female	IV BLZ-21-031	NR(d)	NR(d)	-	-	NR	-	-
**3391**	Female	ICV BLZ-21-031	-	-	-	-	-	-	-
**3392**	Female	ICV BLZ-21-031	NR(d)	NR(d)	-	-	NR	-	-
**3393**	Female	ICV BLZ-21-031	-	-	-	-	NR	-	-
**3394**	Female	ICV BLZ-21-019	-	+	-	-	-	-	-
**3395**	Female	ICV BLZ-21-019	-	(+)	-	-	NR	-	-
**3396**	Female	IV BLZ-21-019	+++	++++	-	-	-	(+)	(+)
**3397**	Female	IV BLZ-21-019	-	++	-	-	NR	-	-
**3398**	Female	IV BLZ-21-019	++	+++	-	-	NR	(+)	(+)
**3399**	Female	ICV BLZ-21-019	(+)	-	-	-	NR	-	-
**3412**	Female	IV BLZ-21-018	+++	++	-	-	-	-	-
**3413**	Female	IV BLZ-21-018	(+)	++++	-	((+))	NR	-	(+)
**3414**	Female	IV BLZ-21-018	+	++	-	-	NR	((+))	(+)
**3415**	Female	ICV BLZ-21-018	-	(+)	-	-	-	-	-
**3416**	Female	ICV BLZ-21-018	-	(+)	-	-	NR	-	-
**3417**	Female	ICV BLZ-21-018	-	-	-	-	NR	-	-
**3418**	Female	ICV BLZ-21-020	-	+	-	-	-	-	-
**3419**	Female	ICV BLZ-21-020	-	(+)	-	-	NR	-	-
**3420**	Female	IV BLZ-21-020	-	(+)	-	-	-	-	-
**3421**	Female	IV BLZ-21-020	++	+++	-	-	NR	(+)	-
**3422**	Female	IV BLZ-21-020	++	+	-	-	NR	((+))	-
**3423**	Female	ICV BLZ-21-020	-	(+)	-	-	NR	-	-
**3400**	Male	IV BLZ-21-031	-	-	-	-	NR	-	-
**3401**	Male	IV BLZ-21-031	NR(d)	-	-	NR	-	-	-
**3402**	Male	IV BLZ-21-031	-	-	NR	NR	NR	-	-
**3403**	Male	ICV BLZ-21-031	-	-	-	-	NR	-	-
**3404**	Male	ICV BLZ-21-031	NR(d)	-	-	NR	-	-	-
**3405**	Male	ICV BLZ-21-031	-	-	NR	NR	NR	-	-
**3406**	Male	ICV BLZ-21-019	-	(+)	-	-	NR	-	-
**3407**	Male	ICV BLZ-21-019	NR(d)	++	-	NR	-	-	-
**3408**	Male	IV BLZ-21-019	++	++	-	-	NR	-	-
**3409**	Male	IV BLZ-21-019	NR(d)	NR(d)	-	NR	NR	(+)	-
**3410**	Male	IV BLZ-21-019	++	++	NR	NR	NR	(+)	(+)
**3411**	Male	ICV BLZ-21-019	-	-	NR	NR	NR	-	-
**3424**	Male	IV BLZ-21-018	(+)	++	-	-	NR	-	-
**3425**	Male	IV BLZ-21-018	NR(d)	+	-	NR	-	-	-
**3427**	Male	ICV BLZ-21-018	-	+	NR	-	NR	-	-
**3428**	Male	ICV BLZ-21-018	NR(d)	(+)	-	NR	-	NR	NR(d)

**3429**	Male	ICV BLZ-21-018	-	-	-	NR	NR	-	-
**3430**	Male	ICV BLZ-21-020	-	++	-	-	-	-	-
**3432**	Male	IV BLZ-21-020	+++	+++	-	-	NR	-	-
**3433**	Male	IV BLZ-21-020	NR(d)	+++	-	NR	-	-	-
**3434**	Male	IV BLZ-21-020	+	+++	NR	NR	NR	(+)	-
**3435**	Male	ICV BLZ-21-020	NR(d)	+	-	NR	NR	-	-

*Tissue for #3389 (IV BLZ-21-031) was left outside the freezer overnight and discarded. Mouse #3431 (ICV, BLZ-21-020) died 3 days after surgery, mouse #3426 accidentally received ICV and IV injections and was removed from analysis. Heart and liver tissue for #3409 was not usable due to complications with lysis. One set of samples for heart could not be determined due to technical failure and lack of sufficient sample. Lung tissue lysis did not yield sufficient lysate for #3428.

Supplementary Table 1 (related to Figure 2): Qualitative FMRP expression in diverse tissues. *[+]*: expression was detected, parentheses and number of + indicate expression levels with more plus signs indicating higher levels of protein expression, and parentheses indicating less expression, e.g.: ((+)) barely detectable; *[-]*: gel was run and no expression detected; *NR*: sample not run on gel (intentional); *NR(d)*: not run on gel due to missing sample.

Supplementary Table 2: Sample Sizes and Statistics of Figs 3, 4 and S2

Fig.	Assay Measurement	Sample Size	Statistical Test	Statistics	Post hoc Tests	
					pair	p-value
3B	Audiogenic Seizure AGS Score	n(vehicle)=16<br>n(AAV/PGK)=10<br>n(AAV/CAG)=15<br>n(AAV/CAG-WPREdel)=11	Kruskal-Wallis test with Dunn's post hoc	H=8.111<br>*p=0.044	veh-PGK<br>veh-CAG<br>veh-CAGdel	p=0.088<br>p>0.999<br>*p=0.047
3C	Audiogenic Seizure % Seizures	n(vehicle)=16<br>n(AAV/PGK)=10<br>n(AAV/CAG)=15<br>n(AAV/CAG-WPREdel)=11	Fisher's exact tests	N/A	veh-PGK<br>veh-CAG<br>veh-CAGdel	p=0.218<br>p=0.722<br>*p=0.042
3C	Audiogenic Seizure % Deaths	n(vehicle)=16<br>n(AAV/PGK)=10<br>n(AAV/CAG)=15<br>n(AAV/CAG-WPREdel)=11	Fisher's exact tests	N/A	veh-PGK<br>veh-CAG<br>veh-CAGdel	p=0.399<br>p=0.716<br>*p=0.022
3E	RNA Quantification	<u>Cerebellum</u><br>n(vehicle)=7<br>n(AAV/CAG)=9<br>n(AAV/CAG-WPREdel)=10<br><br><u>Cortex</u><br>n(vehicle)=8<br>n(AAV/CAG)=8<br>n(AAV/CAG-WPREdel)=10<br><br><u>Hippocampus</u><br>n(vehicle)=5<br>n(AAV/CAG)=4<br>n(AAV/CAG-WPREdel)=4<br><br><u>Midbrain</u><br>n(vehicle)=5<br>n(AAV/CAG)=9<br>n(AAV/CAG-WPREdel)=9<br><br><u>Liver</u><br>n(vehicle)=8<br>n(AAV/CAG)=9<br>n(AAV/CAG-WPREdel)=9	Two-way ANOVA with Dunnett's post hoc (all comparisons within one organ)	<u>Organ:</u><br>F(4,99)=6.2,p=0.0002<br><br><u>Viral vector:</u><br>F(2,99)=16.8,p<0.0001<br><br><u>Interaction:</u><br>F(8,99)=4.2,p=0.0002	ns p>0.7<br>***p=0.0005<br>****p<0.0001	
S2A	Audiogenic Seizure AGS Score	n(vehicle)=16/15<br>n(AAV/PGK)=10/8<br>n(AAV/CAG)=15/12	Two-way ANOVA	<u>Leak:</u><br>F(1,87)=0.1,p=0.75<br><br><u>Virus:</u><br>F(3,87)=16.4,p=0.0009	N/A	

		n(AAV/CAG-WPREdel)=11/8<br><br>[with/without backflow samples]		Interaction:<br>F(3,87)=0.06,p=0.98	
S2 B	Audiogenic Seizure<br>% Seizures	n(vehicle)=16/15<br>n(AAV/PGK)=10/8<br>n(AAV/CAG)=15/12<br>n(AAV/CAG-WPREdel)=11/8<br><br>[with/without leak samples]	Two-way ANOVA	Leak:<br>F(1,3)=0.85,p=0.43<br><br>Viral vector:<br>F(3,3)=47.4,p=0.005<br><br>Interaction:<br>N/A	N/A
S2 C	Audiogenic Seizure<br>% Deaths	n(vehicle)=16/15<br>n(AAV/PGK)=10/8<br>n(AAV/CAG)=15/12<br>n(AAV/CAG-WPREdel)=11/8<br><br>[with/without leak samples]	Two-way ANOVA	Leak:<br>F(1,3)=0.26,p=0.64<br><br>Viral vector:<br>F(3,3)=78.79,p=0.0024<br><br>Interaction:<br>N/A	N/A
S2 D	RNA Quantification	Cerebellum<br>n(vehicle)=6<br>n(AAV/CAG)=8<br>n(AAV/CAG-WPREdel)=8<br><br>Cortex<br>n(vehicle)=7<br>n(AAV/CAG)=8<br>n(AAV/CAG-WPREdel)=8<br><br>Hippocampus<br>n(vehicle)=5<br>n(AAV/CAG)=4<br>n(AAV/CAG-WPREdel)=3<br><br>Midbrain<br>n(vehicle)=4<br>n(AAV/CAG)=8<br>n(AAV/CAG-WPREdel)=7<br><br>Liver<br>n(vehicle)=7<br>n(AAV/CAG)=8<br>n(AAV/CAG-WPREdel)=7	Two-way ANOVA with Tukey's post hoc (all comparisons within one organ)	Organ:<br>F(4,82)=4.9,p=0.0013<br><br>Viral vector:<br>F(2,82)=13.6,p<0.0001<br><br>Interaction:<br>F(8,82)=3.5,p=0.0015	ns p>0.7<br>**p=0.0032<br>***p=0.0001<br>****p<0.0001

4B	Audiogenic Seizure AGS Score	n(vehicle)=12 n(AAV/PGK)=12 n(AAV/CAG)=11 n(AAV/CAG-WPREdel)=12	Kruskal-Wallis test with Dunn's post hoc	H=6.5 p=0.090	veh-PGK veh-CAG veh-CAGdel	p>0.999 *p=0.038 p=0.652
4C	Audiogenic Seizure % Seizures	n(vehicle)=12 n(AAV/PGK)=12 n(AAV/CAG)=11 n(AAV/CAG-WPREdel)=12	Fisher's exact tests	N/A	veh-PGK veh-CAG veh-CAGdel	p>0.999 *p=0.036 p=0.64
4D	Audiogenic Seizure % Deaths	n(vehicle)=12 n(AAV/PGK)=12 n(AAV/CAG)=11 n(AAV/CAG-WPREdel)=12	Fisher's exact tests	N/A	veh-PGK veh-CAG veh-CAGdel	p=0.371 *p=0.012 p=0.193
4E	Open Field Distance traveled	n(vehicle)=12 n(AAV/PGK)=12 n(AAV/CAG)=11 n(AAV/CAG-WPREdel)=12	Welch's ANOVA with Dunnett's post hoc test	W(3, 22.1)=6.1, p=0.004	veh-PGK veh-CAG veh-CAGdel	p=0.412 *p=0.011 *p=0.042
4F	RNA Quantification	Brain n(vehicle)=12 n(AAV/CAG)=11 n(AAV/CAG-WPREdel)=10 Liver n(vehicle)=11 n(AAV/CAG)=10 n(AAV/CAG-WPREdel)=12	Two-way ANOVA with Tukey's post hoc (all comparisons within one organ)	Organ: F(1,60)=14.77,p=0.0003 Viral vector: F(2,60)=36,p<0.0001 Interaction: F(2,60)=9.6,p=0.0002	**p=0.009 ****p<0.0001	

Supplementary Table 3: Sample Sizes and Statistics for Figs 5, 6 and S3

Fig.	Assay	Sample Size	Statistical Test	Statistics	Post hoc Tests
5B	Weight ICV weekly weight	WT ICV Vehicle = 14<br>KO ICV Vehicle = 13<br>KO ICV LD = 11<br>KO ICV HD = 13	Mixed effects model	Week:<br>F(2.782,109.6)=645.4, p<0.0001<br><br>Group:<br>F(3,50)=0.43,p=0.73<br><br>Interaction:<br>F(8.347,109.6)=0.99,p=0.45	N/A
5C	Weight IV weekly weight	WT IV IS Vehicle = 11<br>KO IV IS Vehicle = 10<br>KO IV MD = 10<br>KO IV HD = 8<br>KO IV HD IS = 9	Mixed effects model	Week:<br>F(3.273,117.8)=392.7, p<0.0001<br><br>Group:<br>F(4,44)=0.47,p=0.76<br><br>Interaction:<br>F(13.09,117.8)=0.76,p=0.71	N/A
5D	Survival ICV	WT ICV Vehicle = 14<br>KO ICV Vehicle = 13<br>KO ICV LD = 11<br>KO ICV HD = 13	Logrank (Mantel-Cox) test	$X^2(3)=2.723$, p=0.436	N/A
5E	Survival IV	WT IV IS Vehicle = 11<br>KO IV IS Vehicle = 10<br>KO IV MD = 10<br>KO IV HD = 8<br>KO IV HD IS = 9	Logrank (Mantel-Cox) test	$X^2(4)=23.14$, p=0.0001	N/A
5F	Biodistribution ICV vector copies	WT ICV Vehicle = 12<br>KO ICV Vehicle = 9<br>KO ICV LD = 7<br>KO ICV HD = 5	Mixed effects model with Tukey's post hoc tests	Organ:<br>F(1.029,29.06)=26.14, p<0.0001<br><br>Group:<br>F(3,29)=9.79, p=0.0001<br><br>Interaction:<br>F(3.087,29.06)=8.83, p=0.0002	no significant differences between HD and LD in any organ<br><br>p<0.05 for LD cortex vs all other brain regions
5G	Biodistribution IV vector copies	WT IV IS Vehicle = 7<br>KO IV IS Vehicle = 5<br>KO IV MD = 5<br>KO IV HD = 1<br>KO IV HD IS = 3	Mixed effects model with Tukey's post hoc tests	Organ:<br>F(1,16)=36.89, p<0.0001<br><br>Group:<br>F(4,16)=14.12, p<0.0001<br><br>Interaction:<br>F(4,16)=14.23, p<0.0001	no significant differences between different doses in any organ, or between organs for specific doses

6A	Digging Assay ICV<br>time spent digging	WT ICV Vehicle = 10<br>KO ICV Vehicle = 8<br>KO ICV LD = 10<br>KO ICV HD = 10	RM Two-Way ANOVA with Tukey's post hoc tests	Nest y/n:<br>F(1,34)=46.12,p<0.0001<br><br>Group:<br>F(3,34)=3.27,p=0.033<br><br>Interaction:<br>F(3,34)=1.5,p=0.23	p(ns)>0.1<br>**p=0.003
6B	Digging Assay IV<br>time spent digging	WT IV IS Vehicle = 9<br>KO IV IS Vehicle = 7<br>KO IV MD = 8<br>KO IV HD = 3<br>KO IV HD IS = 8	RM Two-Way ANOVA with Tukey's post hoc tests	Nest y/n:<br>F(1,30)=75.04,p<0.0001<br><br>Group:<br>F(4,30)=2.15,p=0.099<br><br>Interaction:<br>F(4,30)=3.39,p=0.021	p(ns)>0.09<br>*p=0.017<br>**p(veh-MD)=0.0051<br>**p(veh-HD+IS)=0.0015
6C	Overall Activity<br>total time ambulatory	WT ICV Vehicle = 14<br>KO ICV Vehicle = 13<br>KO ICV LD = 11<br>KO ICV HD = 13	RM Two-Way ANOVA with Dunnett's post hoc tests	Interval:<br>F(6.529,306.9)=70.66, p<0.0001<br><br>Group:<br>F(3,47)=1.68,p=0.18<br><br>Interaction:<br>F(33,517)=1.35,p=0.10	*p(wt veh – KO veh)=0.043
6D	Overall Activity<br>time in center	WT ICV Vehicle = 14<br>KO ICV Vehicle = 13<br>KO ICV LD = 11<br>KO ICV HD = 13	RM Two-Way ANOVA with Dunnett's post hoc tests	Interval:<br>F(5.838,274.4)=2.015, p=0.066<br><br>Group:<br>F(3,47)=1.88,p=0.15<br><br>Interaction:<br>F(33,517)=1.23,p=0.18	*p<0.05
6E	Overall Activity<br>total time ambulatory	WT ICV Vehicle = 14<br>KO ICV Vehicle = 13<br>KO ICV LD = 11<br>KO ICV HD = 13	One-Way ANOVA with Dunnett's post hoc tests	F(3,47)=1.68, p=0.184	p(WT-FXS Veh)=0.083<br>p(WT-FXS LD)=0.41<br>p(WT-FXS HD)=0.45
6F	Overall Activity<br>time in center	WT ICV Vehicle = 14<br>KO ICV Vehicle = 13<br>KO ICV LD = 11<br>KO ICV HD = 13	One-Way ANOVA with Dunnett's post hoc tests	F(3,47)=1.88, p=0.147	WT -FXS Veh<br>WT-FXS LD<br>WT-FXS HD<br>p=0.088<br><br>p=0.55<br><br>p=0.17
6G	Overall Activity<br>total time ambulatory	WT IV IS Vehicle = 11<br>KO IV IS Vehicle = 10<br>KO IV MD = 9<br>KO IV HD = 2<br>KO IV HD IS = 7	RM Two-Way ANOVA with Dunnett's post hoc tests	Interval:<br>F(6.802,231.3)=26.68, p<0.0001<br><br>Group:<br>F(4,34)=3.43,p=0.019<br><br>Interaction:<br>F(44,374)=0.88,p=0.69	*p<0.05
6H	Overall Activity<br>time in center	WT IV IS Vehicle = 11	RM Two-Way ANOVA with	Interval:<br>F(6.134,208.5)=1.94,p=0.074	*p<0.05<br>**p<0.01<br>***p=0.0003

		KO IV IS Vehicle = 10<br>KO IV MD = 9<br>KO IV HD = 2<br>KO IV HD IS = 7	Dunnett's post hoc tests	<u>Group:</u><br>F(4,34)=6.75,p=0.0004<br><br><u>Interaction:</u><br>F(44,374)=1.08,p=0.35	
6I	Overall Activity total time ambulatory	WT IV IS Vehicle = 11<br>KO IV IS Vehicle = 10<br>KO IV MD = 9<br>KO IV HD = 2<br>KO IV HD IS = 7	One-Way ANOVA with Dunnett's post hoc tests	F(4,34)=3.43, p=0.19	p(WT- FXS Veh)=0.006<br>p(WT-FXS MD)=0.53<br>p(WT-FXS HD)=0.61<br>p(WT-FXS HD IS)=0.055
6J	Overall Activity time in center	WT IV IS Vehicle = 11<br>KO IV IS Vehicle = 10<br>KO IV MD = 9<br>KO IV HD = 2<br>KO IV HD IS = 7	One-Way ANOVA with Dunnett's post hoc tests	F(4,34)=6.75, p=0.0004	p(WT- FXS Veh)<0.0001<br>p(WT-FXS MD)=0.014<br>p(WT-FXS HD)=0.66<br>p(WT-FXS HD IS)=0.004
S3C	B-cell depletion 4 and 8 weeks after anti-CD20 treatment	no IS, 4wk = 10<br>+ IS, 4wk = 5<br>No IS, 8wk = 13<br>+IS, 8wk = 7	Mixed effects model with Fisher's LSD	<u>Time:</u><br>F(1,12)=12.14, p=0.0045<br><br><u>Immunosuppression:</u><br>F(1,19)=31.55, p<0.0001<br><br><u>Interaction:</u><br>F(1,12)=18.52, p=0.001	****p<0.0001<br>***p=0.0004

Supplementary Table 4A: Sample Sizes and Statistics for Figs 7B,C and S9

Fig.	Assay Measurement	Sample Size	Statistical Test	Statistics
7B	Weight<br>weekly weight gain	WT ICV Vehicle = 13<br>KO ICV Vehicle = 11<br>ICV HD = 11<br>IV HD = 11<br>ICV/IV LD = 11<br>ICV HD/IV LD= 10<br>ICV LD/IV HD= 10	Mixed effects model	Week:<br>F(1.577,100.2)=244.2 p<0.0001<br>Group:<br>F(6,71)=0.76, p=0.60<br>Interaction:<br>F(9.463,100.2)=1.079 p=0.39
7C	Survival	WT ICV Vehicle = 13<br>KO ICV Vehicle = 11<br>ICV HD = 11<br>IV HD = 11<br>ICV/IV LD = 11<br>ICV HD/IV LD= 10<br>ICV LD/IV HD= 10	Logrank (Mantel-Cox) test	$X^2(6)=11.62$, $p=0.071$
S9	Nest Removal time spent digging	WT ICV Vehicle = 13<br>KO ICV Vehicle = 11<br>ICV HD = 11<br>IV HD = 12<br>ICV/IV LD = 10<br>ICV HD/IV LD= 10<br>ICV LD/IV HD= 9	RM Two-Way ANOVA	Nest y/n:<br>F(1,69)=219.2,p<0.0001<br>Group:<br>F(6,69)=0.86,p=0.529<br>Interaction:<br>F(6,69)=1.57,p=0.168

Supplementary Table 4B: Sample Sizes and Statistics for Figs 7E,F

Fig.	Assay<br>Measurement	Sample Size
7E+F	MEA<br>EEG recordings	WT ICV Vehicle = 15<br>KO ICV Vehicle = 16<br>ICV HD = 8<br>IV HD = 9<br>ICV/IV LD = 8<br>ICV HD/IV LD= 7<br>ICV LD/IV HD= 9

ANOVA with Dunnett's post hoc tests (Fig. 7E)

term	estimate	statistic	df	df.residual	mse	p.value
Group	.118	47.43	6	2,124	0.03	< .001
Chan	.085	6.83	29	2,124	0.03	< .001
**contrast**	**estimate**	**conf.int**	**statistic**	**df**	**adj.p.value**	
WT|ICV - KO|ICV	-0.10	[-0.12, -0.07]	-9.22	2124	< .001	
KO|HD-ICV - KO|ICV	-0.08	[-0.11, -0.04]	-6.09	2124	< .001	
KO|HD-IV - KO|ICV	0.05	[0.02, 0.08]	4.11	2124	< .001	
KO|LD-IV+LD-ICV - KO|ICV	-0.06	[-0.09, -0.02]	-4.50	2124	< .001	
KO|HD-IV+LD-ICV - KO|ICV	0.04	[0.01, 0.07]	3.02	2124	.014	
KO|LD-IV+HD-ICV - KO|ICV	0.05	[0.02, 0.08]	3.96	2124	< .001	

Supplementary Table 5: Sample Sizes and Statistics of Figs 7G-I

Fig. 7G	Test	Sample Sizes	Statistics
	Mixed-effects model with Tukey's post hoc tests	WT Veh=6<br>KO Veh=6<br>KO ICV HD=5<br>KO IV HD=5<br>KO ICV/IV LD=5<br>KO ICV HD/IV LD=5<br>KO ICV LD/IV HD=4	Organ<br>F(1.017,28.99)=132.9, p<0.0001<br><br>Group<br>F(6, 29)=35.22, p<0.0001<br><br>Interaction<br>F(6.103,28.99)=38.8, p<0.0001
	**Tissue**	**Significant Pairwise Comparisons**	**p value**
	**Hippocampus**	N/A	none significant
	**Midbrain**	WT Vehicle vs. HD ICV	0.0103
		KO Vehicle vs. HD ICV	0.0103
	**Cerebellum**	WT Vehicle vs. HD ICV + LD IV	0.0123
		KO Vehicle vs. HD ICV + LD IV	0.0123
		HD ICV vs. HD ICV + LD IV	0.0123
		LD ICV + LD IV vs. HD ICV + LD IV	0.0079
	**Cortex**	WT Vehicle vs. HD IV	0.0452
		KO Vehicle vs. HD IV	0.0452
	**Liver**	WT Vehicle vs. HD IV	0.0187
		WT Vehicle vs. LD ICV + HD IV	0.0281
		KO Vehicle vs. HD IV	0.0187
		KO Vehicle vs. LD ICV + HD IV	0.0281
		HD ICV vs. HD IV	0.0191
		HD ICV vs. LD ICV + HD IV	0.0286
		HD IV vs. LD ICV + LD IV	0.0237
		HD IV vs. HD ICV + LD IV	0.0267
		LD ICV + LD IV vs. LD ICV + HD IV	0.0299
		HD ICV + LD IV vs. LD ICV + HD IV	0.0354
**Fig. 7H**	**Test**	**Sample Sizes**	**Statistics**
	Mixed-effects model with Tukey's post hoc tests	Hippocampus<br>WT Veh=4<br>KO Veh=2<br>KO ICV HD=3<br>KO IV HD=2<br>KO ICV/IV LD=2<br>KO ICV HD/IV LD=4<br>KO ICV LD/IV HD=1<br>Midbrain<br>WT Veh=6<br>KO Veh=5<br>KO ICV HD=4<br>KO IV HD=5<br>KO ICV/IV LD=4<br>KO ICV HD/IV LD=4<br>KO ICV LD/IV HD=5<br>Cerebellum, Cortex, Liver<br>WT Veh=6<br>KO Veh=5<br>KO ICV HD=5<br>KO IV HD=5	Organ<br>F(1.936,45.02)=24.32, p<0.0001<br><br>Group<br>F(6, 28)=5.15, p=0.0011<br><br>Interaction<br>F(11.62,45.02)=6.46, p<0.0001

		KO ICV/IV LD=5<br>KO ICV HD/IV LD=4<br>KO ICV LD/IV HD=5	
	**Tissue**	**Significant Pairwise Comparisons**	**p value**
	**Hippocampus**	N/A	none significant
	**Midbrain**	WT Vehicle vs. HD IV	0.0061
		KO Vehicle vs. HD IV	0.0061
	**Cerebellum**	WT Vehicle vs. LD ICV + HD IV	0.0499
		KO Vehicle vs. LD ICV + HD IV	0.0499
	**Cortex**	WT Vehicle vs. HD ICV	0.0192
		WT Vehicle vs. HD IV	0.0003
		KO Vehicle vs. HD ICV	0.0192
		KO Vehicle vs. HD IV	0.0003
		HD ICV vs. HD IV	0.0343
	**Liver**	WT Vehicle vs. LD ICV + LD IV	0.0015
		WT Vehicle vs. LD ICV + HD IV	0.0086
		KO Vehicle vs. LD ICV + LD IV	0.0015
		KO Vehicle vs. LD ICV + HD IV	0.0086
		HD ICV vs. HD IV	0.0007
		HD ICV vs. HD ICV + LD IV	0.0086
**Fig. 7I**	**Test**	**Sample Sizes**	**Statistics**
	Mixed-effects model with Tukey's post hoc tests	<u>Hippocampus, Cerebellum</u><br>KO Veh=5<br>KO ICV HD=5<br>KO IV HD=6<br>KO ICV/IV LD=3<br>KO ICV HD/IV LD=5<br>KO ICV LD/IV HD=4<br><u>Midbrain</u><br>KO Veh=4<br>KO ICV HD=5<br>KO IV HD=6<br>KO ICV/IV LD=3<br>KO ICV HD/IV LD=5<br>KO ICV LD/IV HD=4<br><u>Cerebellum, Cortex, Liver</u><br>KO Veh=5<br>KO ICV HD=5<br>KO IV HD=6<br>KO ICV/IV LD=3<br>KO ICV HD/IV LD=5<br>KO ICV LD/IV HD=4	<u>Brain Region</u><br>$F(1.06,22.25)=11.19$, $p=0.0025$<br><br><u>Group</u><br>$F(5,23)=7.49$, $p=0.0003$<br><br><u>Interaction</u><br>$F(5.3,22.25)=7.74$, $p=0.0002$
	**Tissue**	**Significant Pairwise Comparisons**	**p value**
	**Hippocampus**	N/A	none significant
	**Midbrain**	N/A	none significant
	**Cerebellum**	N/A	none significant
	**Cortex**	KO Vehicle vs. HD ICV + LD IV	0.0371
		HD IV vs. HD ICV + LD IV	0.0428
	**Hippocampus**	N/A	none significant
	**Midbrain**	N/A	none significant
	**Cerebellum**	N/A	none significant
	**Cortex**	KO Vehicle vs. HD ICV + LD IV	0.0371
		HD IV vs. HD ICV + LD IV	0.0428

Supplementary Methods

The following behavioral analyses were performed but results are only reported in the Supplementary Information due to lack of baseline effects (related to Figures 5 and 6 and Supplementary Figures 5-9).

Nesting Behavior

Fresh cages for each mouse were prepared with water bottle and cage card in holder labelled "experiment in progress, please do not move." The cages were otherwise empty (so, no mouse house or food on the floor). Five nestlets were weighed and weights were recorded in notebook and on cage card. A pyramid in the left middle of the cage with four nestlets at the base and one nestlet on top was created. The mouse was placed in the cage, and the cage was put back on the rack. After two hours and with as little disturbance to the mouse as possible, a photo of the nest was taken. After 24 hours, a photo of nest was taken and the amount of unshredded material was weighed. The percentage of nestlet torn was calculated and a rating to each nest was assigned to each nest based on "Assessing nest building in mice" by R. Deacon (Deacon, 2006).

Social Preference

Mice were brought into the test room 30 minutes prior to testing for habituation. Test animals were placed in the center compartment after habituation, and the test software automatically began recording once the animal moved. After a 10-minute container habituation, the animal was removed and the barriers to the end compartments where the "stranger" animals were located were removed. For the Stranger test, inanimate objects were placed on each end compartment, and the test mouse was placed in the center. The animal's movements were tracked for 10 minutes, before the test animal was removed and placed back into its home cage for 5 minutes. A stranger mouse was then placed in one of the ends of the container, and a

different inanimate object was placed in the other side of the container. The test mouse was placed again in the center of the container and movement was recorded for 10 more minutes. Mice that failed to explore both compartments during the stranger test were excluded.

Morris Water Maze

A 150 cm diameter, 51 cm high tank with platforms submerged 1-1.5 cm below water level was used. There were 3 phases to MWM, ACQ, REV, and Cued phase. If a mouse did not find the platform within its allocated time, it was placed on the platform for 5s. Data were collected automatically with video tracking software (Anymaze, Stoelting Company, Wood Dale, IL). The training phase was a 6 trial cued training, which orientated the mice to the platform with a visual indicator showing where the platform was located in the tank. Mice that were unable to find the platform after being retested were excluded from the water maze portion of the study. The first phase of MWM was the ACQ phase which was 4 trials a day for 5 days with a probe trial on the 6th day. The platform was submerged with no visual indicator, and there were distal cues positioned on the north wall. The second phase of MWM was the REV phase which started 24 hours after the completion of the ACQ trials. The REV phase was 4 trials a day for 5 day with a probe trial on the 6th day, with a slightly smaller platform but everything else remained unchanged, and the start position reversed (southwest in phase 1 and northeast in phase 2). The final phase was the Cued phase, which started 24 hours after the completion of the REV phase. The Cued phase was 4 trials over 2 days, where the platform was submerged and a cue (orange ball) was attached to the platform.
